# Body Composition and Risk of Vascular‐Metabolic Mortality Risk in 113 000 Mexican Men and Women Without Prior Chronic Disease

**DOI:** 10.1161/JAHA.122.028263

**Published:** 2023-01-25

**Authors:** Louisa Gnatiuc Friedrichs, Rachel Wade, Jesus Alegre‐Díaz, Raúl Ramirez‐Reyes, Adrián Garcilazo‐Ávila, Carlos Gonzáles‐Carballo, Rogelio Santacruz‐Benitez, Erwin Chiquete, William Herrington, Rory Collins, Richard Peto, Robert Clarke, Jaime Berumen, Jonathan R. Emberson, Pablo Kuri‐Morales, Roberto Tapia‐Conyer

**Affiliations:** ^1^ Clinical Trial Service Unit and Epidemiological Studies Unit, Nuffield Department of Population Health University of Oxford United Kingdom; ^2^ MRC Population Health Research Unit, Nuffield Department of Population Health University of Oxford United Kingdom; ^3^ Experimental Research Unit from the Faculty of Medicine National Autonomous University of Mexico Mexico City Mexico; ^4^ Instituto Nacional de Ciencias Médicas y de la Nutrición, Salvador Zubirán Hospital Mexico City Mexico; ^5^ School of Medicine National Autonomous University of Mexico Mexico City Mexico; ^6^ Instituto Tecnológico y de Estudios Superiores de Monterrey Monterrey Mexico

**Keywords:** body composition, Mexican adults, vascular‐metabolic deaths, Obesity, Women, Epidemiology

## Abstract

**Background:**

Body‐mass index is the sum of fat mass index (FMI) and lean mass index (LMI), which vary by age, sex, and impact on disease outcomes. We investigated the separate and joint relevance of FMI and LMI with vascular‐metabolic causes of death in Mexican adults.

**Methods and Results:**

A total of 113 025 adults aged 35 to 74 years and free from diabetes or other chronic diseases when recruited into the Mexico City Prospective Study were followed for 19 years. Cox models estimated sex‐specific death rate ratios from vascular‐metabolic causes after adjustment for confounders and exclusion of the first 5 years of follow‐up. To account for the strong correlation between FMI and LMI, additional models estimated rate ratios associated with “residual FMI” and “residual LMI” (ie, the residuals from linear regression analyses of FMI on LMI, or vice versa). In both sexes, higher FMI and LMI were associated with higher risks of vascular‐metabolic mortality. For a given (ie, fixed) level of LMI, the rate ratio (95% CI) for vascular‐metabolic mortality per 1 kg/m^2^ higher residual FMI strengthened and was higher in women (1.52 [1.38–1.68]) than in men (1.19 [1.13–1.25]). By contrast, for a given level of FMI, higher residual LMI was inversely associated with vascular‐metabolic mortality (rate ratio per 1 kg/m^2^ 0.67 [0.56–0.80] in women and 0.94 [0.90–0.98] in men).

**Conclusions:**

In this study, higher residual FMI was more strongly associated with vascular‐metabolic mortality in women than in men. Conversely, higher residual LMI was inversely associated with vascular‐metabolic mortality, particularly in women.

**Nonstandard Abbreviations and Acronyms**
FMI: fat mass index
LMI: lean mass index
RR: rate ratio
WHR: waist‐hip ratio

**Clinical Perspective**

**What Is New?**

- This study of Mexican adults demonstrated substantial differences in body composition and risks of premature death from vascular and metabolic causes, with fat and lean mass index (which sum to body mass index) having independent and opposite associations with mortality from such causes.

**What Are the Clinical Implications?**

- The study highlights the importance of components of body mass index for risk of death from vascular‐metabolic causes and suggest that relying on analyses of body mass index alone (which does not distinguish fat mass from lean mass) when assessing associated disease risks may be less informative than the body composition components of body mass index.

Mean levels of adiposity and the prevalence of overweight (body mass index [BMI] ≥25 to <30 kg/m^2^) and obesity (BMI ≥30 kg/m^2^) have increased worldwide in recent decades,^1^ and both have been particularly high in Mexico.^2^ BMI is the widely used measure of general adiposity in research^3^ and clinical practice,^4^ but interpretation of the results of observational studies of associations of BMI with mortality is complicated by J‐shaped associations (even after exclusion of individuals with prior disease and deaths occurring during the first few years of follow‐up).^3^, ^5^, ^6^, ^7^ Variation in lean mass between individuals may confound the associations of fat mass with cause‐specific mortality (and vice versa), and so distinguishing the separate effects of fat and lean mass could have important implications for prevention of vascular‐metabolic diseases.^8^, ^9^

A recent report of an observational analysis of over 0.3 million adults from the UK Biobank demonstrated strong positive associations of fat mass with incident cardiovascular disease in both men and women, whereas for skeletal muscle mass, the positive association was much weaker in men (and curvilinear in women).^10^ Mendelian randomization analyses, also conducted within the UK Biobank, confirmed positive associations of FMI with several cardiovascular diseases (and suggested inverse associations of fat‐free mass index with several diseases).^11^ Similarly, a report on 38 000 men from the Health Professional Follow‐up Study demonstrated strong positive associations of predicted fat mass with vascular mortality (independently of lean mass), but the association for lean mass given fat mass was U‐shaped.^8^ Little is known about the relevance of body composition for vascular‐metabolic mortality in Hispanic populations. In an analysis of 10 000 Mexican‐Americans from the NHANES (National Health and Nutrition Examination Survey), neither fat nor lean mass were significantly associated with all‐cause mortality.^12^

The aims of the present study were to assess the sex‐specific independent relevance of fat mass index (FMI) and lean mass index (LMI) for vascular‐metabolic mortality in a 19‐year follow‐up of 150 000 adults in the MCPS (Mexico City Prospective Study).

## Methods

### Data Availability

Data from the MCPS are available for open‐access data requests to bona fide academic researchers. If you are interested in making such a request, or in collaborating with MCPS investigators on a specific research proposal, please visit www.ctsu.ox.ac.uk/research/mcps where you can download the study's Data and Sample Access Policy in English or Spanish. The data currently available for sharing may also be reviewed on the study's online Data Showcase (https://datashare.ndph.ox.ac.uk/mexico).

### Recruitment of Participants

Between 1995 and 1997, a confidential record of all households within 2 districts of Mexico City (Coyoacán and Iztapalapa) was compiled by census‐style door‐to‐door interviews. Subsequently, between 1998 and 2004, recruitment teams (each comprising 2 or 3 specially trained nurses) visited 112 333 individual households, working systematically through this record, and of those, 106 059 households (response rate 94%) yielded 159 755 residents aged 35 years or older (52 644 men and 107 111 women) who agreed to participate.^13^ Research ethics approval was obtained from the Mexican Ministry of Health, the Mexican National Council of Science and Technology, and the University of Oxford (United Kingdom). All the study participants provided written informed consent.

### Baseline and Follow‐Up Surveys

At the baseline assessment, information on demographic factors (self‐reported age and sex and district of residence), lifestyle factors (eg, smoking status, alcohol intake, physical activity), medical history, and current medication was recorded by interview‐administered questionnaire. Weight, height, and waist and hip circumferences were measured in all participants (to the nearest 0.1 kg or 0.1 cm, respectively, using calibrated electronic scales, stadiometers and nonstretchable tapes). A 10 mL blood sample was collected from each participant, from which glycosylated hemoglobin was measured, using validated high‐performance liquid chromatography methods.^14^ Between 2015 and 2019, a resurvey of 10 143 randomly selected surviving participants from both districts collected repeat questionnaires, physical measurements, and blood and urine samples. Direct measurements of body composition were assessed using calibrated bioelectrical impedance Tanita SC‐240MA scales.

### Predicted Body Composition at Baseline

Total fat mass (in kilograms) was predicted in all participants *as a function* of age, sex, height, weight, and waist circumference measured at recruitment, by applying Mexican‐specific equations derived from the NHANES study.^15^ Total lean mass (in kilograms) was then obtained by subtracting predicted fat mass from total weight. Predicted FMI and LMI were then calculated as predicted fat (or lean) mass divided by the square of height in meters (kg/m^2^). (Thus, FMI plus LMI equaled BMI.) These predicted levels of FMI and LMI were subsequently validated in the subset of participants with direct bioimpedance measures collected at the follow‐up resurvey of randomly‐selected surviving participants.

### Mortality Follow‐Up and Outcomes

Deaths up to January 2021 were identified through probabilistic linkage to the Mexican Electronic Death Registry (*Sistema Epidemiologico y Estadıstico de las Defunciones*), with field validation of a subset confirming the reliability of such matched deaths. The causes of death reported on the death certificates were coded according to the *International Classification of Diseases*, *Tenth Revision* (*ICD‐10*).^16^ Study clinicians then reviewed and, where necessary, recoded for the underlying cause of death (eg, accepting diabetes as the underlying cause only for acute diabetic crises).^17^ Vascular‐metabolic deaths comprised vascular deaths (subdivided as ischemic heart disease, stroke, and other vascular disease) and metabolic deaths (renal, acute diabetic crisis, and hepatobiliary disease; see Table S1).

### Statistical Analysis

To minimize the risk of reverse causality bias of preexisting disease on fat and/or muscle mass (including from poorly controlled diabetes),^18^, ^19^ the analyses excluded participants who reported previously‐diagnosed diabetes, regular use of antidiabetic medication, or with glycosylated hemoglobin ≥6.5% at baseline, as well as participants with a prior diagnosis of angina, myocardial infarction, stroke, cancer, chronic kidney disease, cirrhosis, or emphysema. Furthermore, prospective analyses excluded deaths occurring during the first 5 years of follow‐up (to further reduce the influence of reverse causality bias), and deaths occurring after age 75 years (to minimize possible effects of aging on loss of muscle mass^20^).

Pearson correlation coefficients between predicted markers of fat and lean mass and anthropometric measures of adiposity (eg, BMI and waist‐to‐hip ratio [WHR]), were estimated separately for women and men. Cox regression was used to estimate the associations between FMI and LMI and specific vascular or metabolic causes of death at ages 40 to 74 years, separately in men and women. To investigate the *independent* associations of each of these highly correlated measures of body composition with mortality risk (while avoiding any collinearity problems arising by putting both variables in the same model), analyses were conducted using the sex‐specific *residuals* of each marker obtained by regressing each marker on the other. Specifically, “residual FMI” was obtained from the residuals of a regression of FMI on LMI (indicating the difference between FMI and LMI‐predicted FMI), and “residual LMI” was obtained from the residuals of a regression of LMI on FMI (indicating the difference between LMI and FMI‐predicted LMI).

To assess the shape of the association of body composition with specific causes of death, participants were classified into 4 groups defined by quartiles of the sex‐specific distributions of FMI and LMI (separately for predicted and residual values). All models were adjusted for age at risk (in 5‐year groups), district of residence, highest level of education attained (university or college, high school, elementary school, or other), leisure‐time physical activity (none, up to twice weekly, at least 3 times weekly), tobacco use (never, former, occasional, <10 cigarettes per day, ≥10 cigarettes per day), and alcohol consumption (never, former, current). Log death rate ratios (RRs, estimated by the Cox hazard ratio) and group‐specific 95% CIs were calculated for each group (including the reference group, so that CIs can be used to compare risks in any 2 groups),^21^ separately in women and men. These RRs were subsequently plotted against the sex‐specific mean levels of FMI and LMI in each group. The average mortality RR corresponding to a 1 kg/m^2^ higher (predicted or residual) level of each marker of body composition, was calculated by performing a weighted regression through the 4 sex‐specific log RR estimates (with weights equal to the inverse of the variances of the 4 log RRs). Sensitivity analyses included estimates by strata of confounders (with tests of heterogeneity or trend across levels of each confounder), inclusion of individuals with undiagnosed diabetes at recruitment (ie, no previously‐diagnosed diabetes but glycosylated hemoglobin ≥6.5%), and exclusion of current or former smokers.

To compare the ability of adiposity markers to predict subsequent mortality from particular vascular‐metabolic causes of death, the relative “informativeness” of a given adiposity marker (BMI, FMI, LMI, and WHR) was estimated from the age‐adjusted and confounder‐adjusted χ^2^ statistic relating it to the particular cause (or causes) of death.^22^

Throughout, emphasis was on effect size estimation rather than hypothesis testing (although some statistical tests for trend in log RRs across levels of subgroups are shown in the appendix). All statistical analyses were performed using SAS v9.4 and R v4.1.3 (www.r‐project.org).

## Results

Of 112 333 eligible households visited, 106 059 (94%) yielded a total of 159 755 participants. Of these 159 755, 12 116 (8%) were excluded from the analyses in this article because they were aged 75 years or older at recruitment, a further 6714 (4%) were excluded because of a history of chronic disease other than diabetes, and a further 24 902 (16%) were excluded because of a history of diabetes or because they had glycosylated hemoglobin ≥6.5% at baseline. Of the remaining 116 023 participants, a further 2998 (3%) were excluded because of missing or implausible data, or duplicate participants, leaving 113 025 participants aged 35 to 74 years for the present analyses.

### Baseline Characteristics of Study Participants

The mean (SD) age of participants at baseline was 49 (10) years and 32% were men. A higher proportion of men than women were current smokers (52% versus 25%), took regular leisure‐time physical activity (31% versus 19%), and were educated to university or college level (27% versus 14%; Table 1). In men, mean (SD) BMI was 28.0 (4.1) kg/m^2^, reflecting an FMI of 8.2 (2.2) kg/m^2^ and an LMI of 19.8 (2.1) kg/m^2^. In women, mean (SD) BMI was 29.5 (5.0) kg/m^2^, reflecting an FMI of 12.8 (3.3) kg/m^2^ and an LMI of 16.7 (1.8) kg/m^2^. In both men and women residency in Coyoacán (the wealthier of the 2 districts), university/college education, current smoking, and regular leisure‐time physical activity were more common among those with lower than higher FMI; associations of these factors with LMI were similar but less consistent (Table S2). In both sexes, levels of blood pressure and glycosylated hemoglobin were higher in those with higher FMI and/or LMI.

**Table 1:** Characteristics of 113 025 Participants Aged 35 to 74 at Recruitment, by Sex

	Men (n=36 612, 32%)	Women (n=76 413, 68%)	All (n=113 025)
Age, y	50 (11)	49 (10)	49 (10)
Socioeconomic status and lifestyle behaviors			
Resident of Coyoacán	16 511 (45%)	30 334 (40%)	46 845 (41%)
University/college educated	9973 (27%)	10 597 (14%)	20 570 (18%)
Current smoker	19 056 (52%)	19 202 (25%)	38 258 (34%)
Current drinker	29 185 (80%)	49 760 (65%)	78 945 (70%)
Any regular leisure‐time physical activity	11 483 (31%)	14 683 (19%)	26 166 (23%)
Biological measurements			
Systolic BP, mm Hg	127 (15)	124 (16)	125 (15)
Diastolic BP, mm Hg	84 (10)	82 (10)	83 (10)
Glycosylated hemoglobin, %	5.4 (0.4)	5.5 (0.4)	5.5 (0.4)
Long‐term medication use			
Any antihypertensive	2752 (8%)	9679 (13%)	12 431 (11%)
Any antithrombotic	731 (2%)	1939 (3%)	2670 (2%)
Any lipid‐lowering	150 (<0.5%)	312 (<0.5%)	462 (<0.5%)
Physical measurements			
Height, cm	165 (7)	152 (6)	156 (9)
Weight, kg	76 (13)	68 (12)	71 (13)
BMI, kg/m^2^	28.0 (4.1)	29.5 (5.0)	29.0 (4.8)
Waist circumference, cm	96 (10)	92 (12)	94 (11)
Hip circumference, cm	101 (8)	106 (11)	105 (10)
Waist–hip ratio	0.95 (0.06)	0.87 (0.06)	0.89 (0.07)
Fat mass, kg*	22 (6)	30 (8)	27 (8)
Lean mass, kg†	54 (7)	39 (5)	44 (9)
Fat mass index, kg/m^2^ ‡	8.2 (2.2)	12.8 (3.3)	11.3 (3.7)
Lean mass index, kg/m^2^ §	19.8 (2.1)	16.7 (1.8)	17.7 (2.4)

BMI indicates body mass index; and BP, blood pressure. Mean (SD) or n (column %) shown. Table excludes participants with previously diagnosed diabetes, those without previously diagnosed diabetes but with a glycosylated hemoglobin concentration at recruitment of 6.5% or greater, those with chronic disease (ischemic heart disease, stroke, chronic kidney disease, cirrhosis, cancer, or emphysema) at recruitment, those with missing data on any analysis covariate (sex, district of residence, educational level attained, smoking status, alcohol intake, leisure time physical activity), uncertain follow‐up, or missing or extreme measures of anthropometry: height (cm) <120 or >200, weight (kg) <35 or >250, BMI (kg/m^2^) <18.5 or ≥60, waist circumference (cm) <60 or >180, hip circumference (cm) <70 or >180, waist–hip ratio <0.5 or >1.5.

* Fat mass (kg) estimated using Mexican‐specific equations from the NHANES (National Health and Nutrition Examination Survey) study^18^ (Men: $-18.592-0.009*\mathrm{age}\left(\mathrm{yrs}\right)-0.080*\text{height}\left(\mathrm{cm}\right)+0.226*\text{weight}\left(\mathrm{kg}\right)+0.387*\text{waist circumference}\left(\mathrm{cm}\right)+0.080$, Women: $\begin{array}{c} 11.817+0.041*\mathrm{age}\left(\mathrm{yrs}\right)-0.199* \end{array}$ $\begin{array}{c} \text{height}\left(\mathrm{cm}\right)+0.610*\text{weight}\left(\mathrm{kg}\right)+0.044*\text{waist circumference}\left(\mathrm{cm}\right)+0.388 \end{array}$).

^†^ Lean mass equals total weight minus estimated fat mass.

^‡^ Fat mass index calculated as estimated fat mass (kg) divided by the square of height (m^2^).

^§^ Lean mass index equals BMI minus estimated fat mass index.

**Figure 1:**
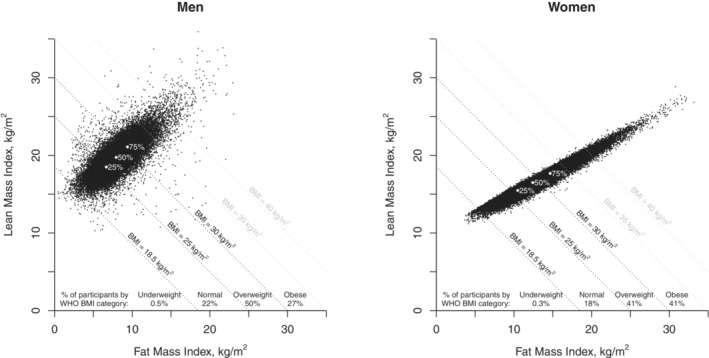
Fat mass index vs lean mass index in men and women aged 35 to 74 years and without diabetes or chronic disease at recruitment. Fat mass index (FMI) and lean mass index (LMI) estimated using Mexican−specific equations from the NHANES study (see footnote to Table 1). Underweight participants (15<= (BMI<18.5) are plotted in this figure for information but excluded from all analyses. The solid white points show, for both men and women, the 25th, 50th, and 75th centiles of the FMI and LMI distributions; these correspond to the cutpoints used to define the 4 main groups in the prospective analyses. BMI indicates body mass index; NHANES, National Health and Nutrition Examination Survey; and WHO, World Health Organization.

FMI and LMI were strongly correlated with each other, particularly in women (Figure 1). In the subset of participants with physical measurements and direct estimates of body composition from bioimpedance measures at resurvey, the correlation between bioimpedance estimated and NHANES‐predicted FMI and LMI was high, particularly for FMI (Figure S1). Baseline FMI increased with age up to 60 years, particularly in women, whereas LMI decreased with age in men and decreased with age only after about age 50 years in women (Figure S2). In both sexes, FMI and LMI were highly correlated with BMI, but the residuals of each value (ie, FMI *given knowledge of* LMI, and vice versa) were only weakly correlated with BMI (Table S3). (By construction, residual FMI was uncorrelated with LMI and residual LMI was uncorrelated with FMI.) FMI was moderately correlated with WHR in men and women, whereas LMI was only weakly correlated with WHR.

### Body Composition and Mortality From Any Vascular‐Metabolic Cause

**Figure 2:**
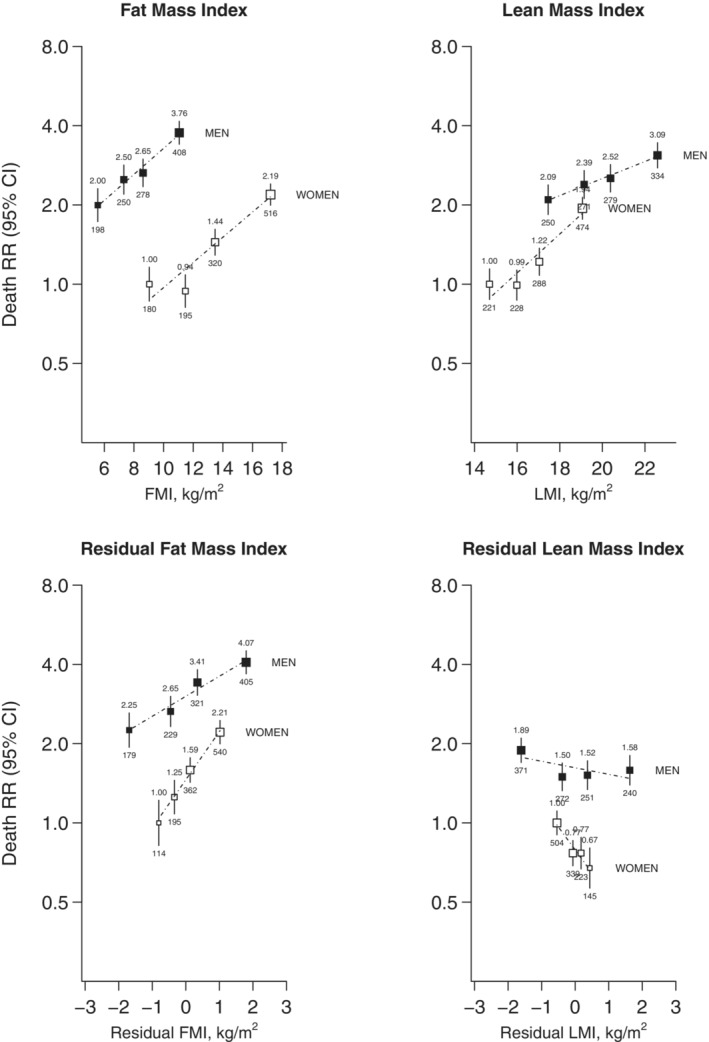
Relevance of FMI and LMI to vascular‐metabolic mortality in men and women at ages 40 to 74 years. Body composition and vascular‐metabolic mortality at ages 40 to 74 years, separately in men and in women (men had about twice the mortality rates of women). Participant exclusions are as in Table 1. In addition, deaths within the first 5 years of follow‐up are excluded to limit reverse causality. The 4 groups shown correspond to sex‐specific quartiles of each distribution. The RRs for the 4 sex‐specific categories were plotted at the mean baseline level in each group, with the vertical lines through each point representing group‐specific 95% CIs (with the area of each plotting symbol proportional to the amount of statistical information). RRs were adjusted for age at risk, district of residence, self‐reported highest level of education attained, leisure‐time physical activity status, smoking status, and alcohol intake. The RR is noted above each vertical line, and the number of deaths below. The average mortality RR per 1 kg/m^2^ higher baseline level (shown by dashed lines on the panels in Figure 1, and tabulated in Table 2) is assessed throughout the full range studied and is calculated by performing a weighted regression through the 4 sex‐specific log RR estimates (with weights equal to the inverse of the variances of the log RRs). For associations that do not appear to be log‐linear, these average RRs may reflect different underlying RRs at different levels of body composition. FMI indicates fat mass index; LMI, lean mass index; and RR, rate ratio.

With participants tracked to January 1, 2021, median duration of follow‐up among survivors was about 19 years. After excluding deaths occurring during the first 5 years of follow‐up, there were 2345 deaths from vascular‐metabolic causes before age 75 years, including 745 from ischemic heart disease, 289 from stroke, 419 from renal or acute diabetic causes, and 549 from hepatobiliary diseases. Overall, the men in the study population had about twice the death rate of the women (Figure 2). In both men and women, FMI and LMI were linearly and positively associated with vascular‐metabolic mortality after adjustment for confounders (Figure 2). Across the ranges of the FMI and LMI values studied, the average RRs (95% CI) for vascular‐metabolic mortality associated with each 1 kg/m^2^ higher FMI were 1.12 (1.09–1.15) in men and 1.12 (1.09–1.14) in women. In contrast for LMI, the RRs (95% CI) associated with 1 kg/m^2^ higher levels were 1.08 (1.04–1.11) in men and 1.19 (1.14–1.23) in women (Table 2). At any given level of LMI, the between‐subject variation in FMI was greatly reduced, particularly in women. Nonetheless, for a given LMI, the RRs (95% CI) associated with a 1 kg/m^2^ higher residual FMI increased, particularly for women (1.19 [1.13–1.25] in men versus 1.52 [1.38–1.68] in women) (Table 2). In contrast at any given level of FMI, higher levels of residual LMI were inversely associated with vascular‐metabolic mortality: the RRs (95% CI) associated with 1 kg/m^2^ higher residual LMI were 0.94 (0.90–0.99) in men and 0.67 (0.56–0.80) in women (Table 2).

**Table 2:** Predicted Body Composition and Cause‐Specific Vascular‐Metabolic Mortality at Ages 40 to 74 Years

Cause of death	Men					Women				
	No. of deaths	Death RR (95% CI) per 1 kg/m^2^ higher level				No. of deaths	Death RR (95% CI) per 1 kg/m^2^ higher level			
		FMI	Residual FMI	LMI	Residual LMI		FMI	Residual FMI	LMI	Residual LMI
Vascular										
Ischemic heart disease	413	1.12 (1.06–1.17)	1.12 (1.04–1.22)	1.12 (1.07–1.18)	1.03 (0.95–1.12)	332	1.09 (1.05–1.13)	1.64 (1.36–1.98)	1.13 (1.06–1.21)	0.58 (0.41–0.82)
Stroke	104	1.20 (1.09–1.33)	1.49 (1.25–1.78)	1.08 (0.97–1.21)	0.80 (0.68–0.95)	185	1.05 (1.00–1.11)	1.50 (1.16–1.94)	1.06 (0.96–1.16)	0.52 (0.32–0.85)
Other vascular	125	1.06 (0.97–1.17)	1.27 (1.09–1.48)	1.00 (0.91–1.10)	0.78 (0.67–0.92)	218	1.09 (1.04–1.14)	1.15 (0.91–1.45)	1.17 (1.08–1.27)	0.98 (0.64–1.50)
Subtotal: any vascular	642	1.12 (1.07–1.16)	1.20 (1.12–1.28)	1.09 (1.05–1.14)	0.94 (0.88–1.01)	735	1.08 (1.06–1.11)	1.45 (1.28–1.65)	1.13 (1.08–1.18)	0.66 (0.52–0.83)
Metabolic										
Renal/acute diabetic crisis	185	1.17 (1.09–1.26)	1.23 (1.09–1.38)	1.10 (1.01–1.19)	0.95 (0.84–1.07)	234	1.18 (1.13–1.24)	1.15 (0.91–1.44)	1.32 (1.21–1.43)	1.57 (1.04–2.35)
Hepatobiliary	307	1.09 (1.03–1.16)	1.15 (1.05–1.26)	1.04 (0.98–1.10)	0.95 (0.86–1.05)	242	1.16 (1.11–1.21)	2.14 (1.71–2.68)	1.26 (1.16–1.36)	0.36 (0.24–0.53)
Subtotal: any metabolic	492	1.12 (1.07–1.17)	1.18 (1.10–1.27)	1.06 (1.01–1.11)	0.95 (0.88–1.02)	476	1.17 (1.13–1.21)	1.62 (1.38–1.90)	1.29 (1.21–1.36)	0.70 (0.53–0.92)
**All vascular‐metabolic**	**1134**	**1.12 (1.09–1.15)**	**1.19 (1.13–1.25)**	**1.08 (1.04–1.11)**	**0.94 (0.90–0.99)**	**1211**	**1.12 (1.09–1.14)**	**1.52 (1.38–1.68)**	**1.19 (1.14–1.23)**	**0.67 (0.56–0.80)**

Conventions, main exclusions, and analyses as per Figure 2, but analyses are for cause‐specific mortality end points (Table S1). Results displayed in the table are the average mortality RR (95% CI) per 1 kg/m^2^ higher baseline level. FMI indicates fat mass index; LMI, lean mass index; and RR, rate ratio.

### Body Composition and Mortality From Specific Vascular‐Metabolic Causes

Figures S3 through S9 show the sex‐specific associations of each marker of body composition with subtypes of vascular‐metabolic mortality as outlined in Table 2. In both men and women, FMI was positively and typically log‐linearly associated with higher risks of each cause of death. After accounting for LMI, the associations of residual FMI with particular vascular‐metabolic causes of death were increased in both men and women for most, but not for all individual causes of death.

In both men and women, LMI was positively associated with the risk of particular vascular‐metabolic causes of death. However, after accounting for FMI, the associations of residual LMI with particular causes of death were reversed for most causes of death, particularly for women. For example, in women the RRs for each 1 kg/m^2^ higher residual LMI were 0.58 (0.41–0.82) for ischemic heart disease mortality, 0.52 (0.32–0.85) for stroke mortality, and 0.70 (0.53–0.92) for death from any metabolic cause.

### Sensitivity Analyses

In both men and women, the associations of FMI, LMI, residual FMI, and residual LMI with vascular‐metabolic mortality risk were highly consistent irrespective of age, smoking, drinking, physical activity, educational achievement, or residential district (Figure S10). In women, however, residual FMI was more strongly and positively associated, and residual LMI more strongly inversely associated, with vascular‐metabolic mortality among those who were better educated. Estimates of cause‐specific mortality were not materially altered when repeated after inclusion of participants with undiagnosed diabetes at baseline or when restricted to never‐smokers (Table S4).

### Comparative Importance of Different Measures of Body Composition

Comparison of the predictive value of a single baseline measure of BMI, WHR, FMI, and LMI for prediction of risk of death from particular vascular‐metabolic causes is shown in Table S5. In men, WHR and FMI were the most informative predictors of vascular‐metabolic mortality (although BMI remained the most informative predictor of ischemic heart disease mortality). In women, FMI was the most informative predictor of vascular‐metabolic mortality (including metabolic mortality and vascular mortality), though the WHR was the most informative predictor of stroke.

## Discussion

This report highlighted substantial differences in body composition and age‐related changes in FMI and LMI among Mexican women and men and also demonstrated striking differences in the associations of different markers of body composition with risk of death from vascular‐metabolic causes. Given LMI, higher levels of FMI were strongly and positively associated with higher risks of death from vascular‐metabolic causes (particularly in women). Conversely, given FMI, higher levels of LMI were inversely associated with vascular‐metabolic mortality, chiefly in women. Indeed, in women, each 1 kg/m^2^ higher *residual* FMI was associated with 52% higher risk of vascular‐metabolic mortality whereas each 1 kg/m^2^ higher *residual* LMI was associated with 33% lower risk of vascular‐metabolic mortality.

### Comparison With Previous Studies

The results of the present study of adults from Mexico City are consistent with those obtained from previous large studies conducted in high‐income populations. In the Health Professional Follow‐up Study of 38 000 men, individuals in the top versus bottom fifth of fat mass had 67% higher risks of vascular mortality after adjustment for LMI and other factors, but the study was restricted to men.^8^ A study of 350 000 participants in UK Biobank (about 50% female) reported that each 1 SD higher bioimpedance‐measured fat mass (about 9 kg) was associated with 20% higher risk of incident cardiovascular disease in men and 25% higher risk in women.^10^ Mendelian randomization analyses (also conducted within the UK Biobank) demonstrated that genetically elevated FMI was associated with higher risks of several cardiovascular diseases, including an 8% higher odds of coronary heart disease (per 1 kg/m^2^ higher FMI) and 38% higher odds of ischemic stroke.^11^ The latter analysis also indicated that higher levels of genetically instrumented LMI was significantly associated with a lower odds of several cardiovascular diseases, including a 25% lower odds of ischemic stroke per 1 kg/m^2^ higher LMI (again, consistent with our sex‐specific estimates for residual LMI). Little is known about the independent relevance of the associations of different measures of body composition in Hispanic populations, but a previous study of 10 000 Mexican‐Americans reported positive associations of dual‐energy X‐ray absorptiometry‐measured FM percentage with mortality from diabetes but not with vascular causes.^12^

### Mechanisms Underlying the Observations

The positive associations of higher levels of FMI with particular vascular‐metabolic causes of death are probably causal. The mechanisms by which FMI causes death from vascular‐metabolic causes likely include higher levels of blood pressure,^23^, ^24^ insulin resistance and glucose intolerance, elevated levels of nonesterified fatty‐acids and procoagulation factors, and reduced clearance of apolipoprotein‐B, triglycerides, and very‐low‐density lipoproteins.^25^, ^26^ However, the causal relevance of the inverse associations of higher levels of LMI with vascular‐metabolic causes of death is less certain. Irrespective of this, however, the findings of the present study provide important new evidence that higher levels of muscle mass may be protective against vascular‐metabolic diseases after accounting for the level of fat mass.^27^, ^28^

### Strengths and Limitations

The chief strengths of the present study included the large number of individuals studied and prolonged duration of follow‐up (including several thousand deaths), exclusion of individuals with a prior history of major chronic diseases, and control of a wide range of potential confounders (albeit the possibility of residual confounding cannot be fully excluded). Although no direct measurements of fat or lean mass were recorded at recruitment, the measures of body composition estimated in the present study were based on externally derived Mexican‐specific prediction equations (using age, sex, weight, height, and waist circumference) and were highly correlated with measures recorded using bioimpedance in a random subset of participants at resurvey. FMI and LMI were highly correlated, so instead of including both measures in the same regression model (which could result in collinearity), we used residual levels of FMI and LMI to estimate their independent relevance for cause‐specific mortality separately in men and women, respectively. Covariates varied in their distributions between men and women, but this did not affect the results because analyses involving covariate adjustment were done separately in men and women. The chief limitation of the present study was the lack of any information on nonfatal diseases. In addition, the present study relied on causes of death listed on the death certificate. However, almost all deaths in Mexico are certified by a doctor and the overall accuracy and quality of certification of causes of death in Mexico is high.^29^ More women than men were recruited into the study (because women were more likely to be at home when the fieldworkers' visit was during standard working hours). However, death rates were higher in men than women, resulting in similar numbers of deaths for the analyses of men compared with women. Response rates in the 2 study districts were high, but the districts themselves are not representative of the overall Mexican population or even the overall Mexico City population. However, although debated,^30^ prospective studies of nonrepresentative cohorts of individuals can provide reliable evidence about the associations of risk factors with disease that are widely generalizable (at least qualitatively).^31^

### Implications of Our Findings

The findings of the present study have important implications for selection of the optimum measure of adiposity for prediction of risk of cardiometabolic diseases. Compared with BMI, the estimates of FMI included in the present report were more informative for prediction of vascular‐metabolic causes of death (particularly for women). Recognition of the differences in the importance of body composition for deaths from vascular‐metabolic causes observed in this report has implications for specific determinants for *optimal high body mass*, with healthy levels of high lean mass for prevention of premature deaths from vascular‐metabolic causes, both in men and in women.

## Conclusions

Overall, the findings of the present study of Mexican adults highlighted the adverse effects of higher levels of fat mass for risk of vascular‐metabolic diseases and also suggested that in this population higher levels of lean mass were inversely associated with such diseases, particularly for women.

## Sources of Funding

The Mexico City Prospective Study has been supported by grants from the Wellcome Trust (058299/Z/99), Mexican Health Ministry, Mexican National Council of Science and Technology, British Heart Foundation, Cancer Research UK, and UK Medical Research Council (MC_UU_00017/2). The funding sources had no input into the design, conduct, and analysis of the study or the decision to submit the article for publication.

## Disclosures

None.

## Supporting information

Tables S1–S5
Figures S1–S10
