# Supplementary material for: Body Composition and Risk of Vascular‐Metabolic Mortality Risk in 113 000 Mexican Men and Women Without Prior Chronic Disease
Source: J Am Heart Assoc. 2023 Jan 25;12(3):e028263. doi: 10.1161/JAHA.122.028263 (PMC9973642; doi:10.1161/JAHA.122.028263)
Supplement: Supplementary file 1 — Tables S1–S5 Figures S1–S10 [file JAH3-12-e028263-s001.pdf]

Supplementary Material

Body composition and risk of vascular-metabolic mortality risk in 113,000 Mexican men and women without prior chronic disease

Supplementary figures

Pg

1:	NHANES−estimated vs bio−impedance measured body composition, in men and women aged <90 years at resurvey	2
2:	Predicted body composition by age and sex in those aged 35−74 years and without diabetes or chronic disease at recruitment	3
3.	Relevance of FMI and LMI to IHD mortality, in men and women, at ages 40−74 years	4
4.	Relevance of FMI and LMI to stroke mortality, in men and women, at ages 40−74 years	5
5:	Relevance of FMI and LMI to other vascular mortality, in men and women, at ages 40−74 years	6
6.	Relevance of FMI and LMI to any vascular mortality, in men and women, at ages 40−74 years	7
7:	Relevance of FMI and LMI to renal/acute diabetic mortality, in men and women, at ages 40−74 years	8
8:	Relevance of FMI and LMI to hepatobiliary mortality, in men and women, at ages 40−74 years	9
9.	Relevance of FMI and LMI to any metabolic mortality, in men and women, at ages 40−74 years	10
10a:	Relevance of predicted body composition to vascular−metabolic mortality in men, by levels of confounders	11
10b:	Relevance of predicted body composition to vascular−metabolic mortality in women, by levels of confounders	12

Supplementary tables

1:	Numbers of deaths at ages 40-74 by underlying cause (ICD-10 code)	13
2a:	Characteristics of 113 025 participants aged 35-74 at recruitment, by sex and fat mass index	14
2b:	Characteristics of 113 025 participants aged 35-74 at recruitment, by sex and lean mass index	15
3.	Correlation of markers of body composition in those aged 35-74 years at recruitment and eligible for the prospective analyses	16
4:	Predicted body composition and cause-specific vascular-metabolic mortality at ages 40-74 years - sensitivity analyses	17
5:	Comparison of the 'informativeness' of different body composition indices for cause-specific mortality rates at ages 40-74 years	18

Webfigure 1: NHANES−estimated vs bio−impedance measured body composition, in men and women aged <90 years at resurvey

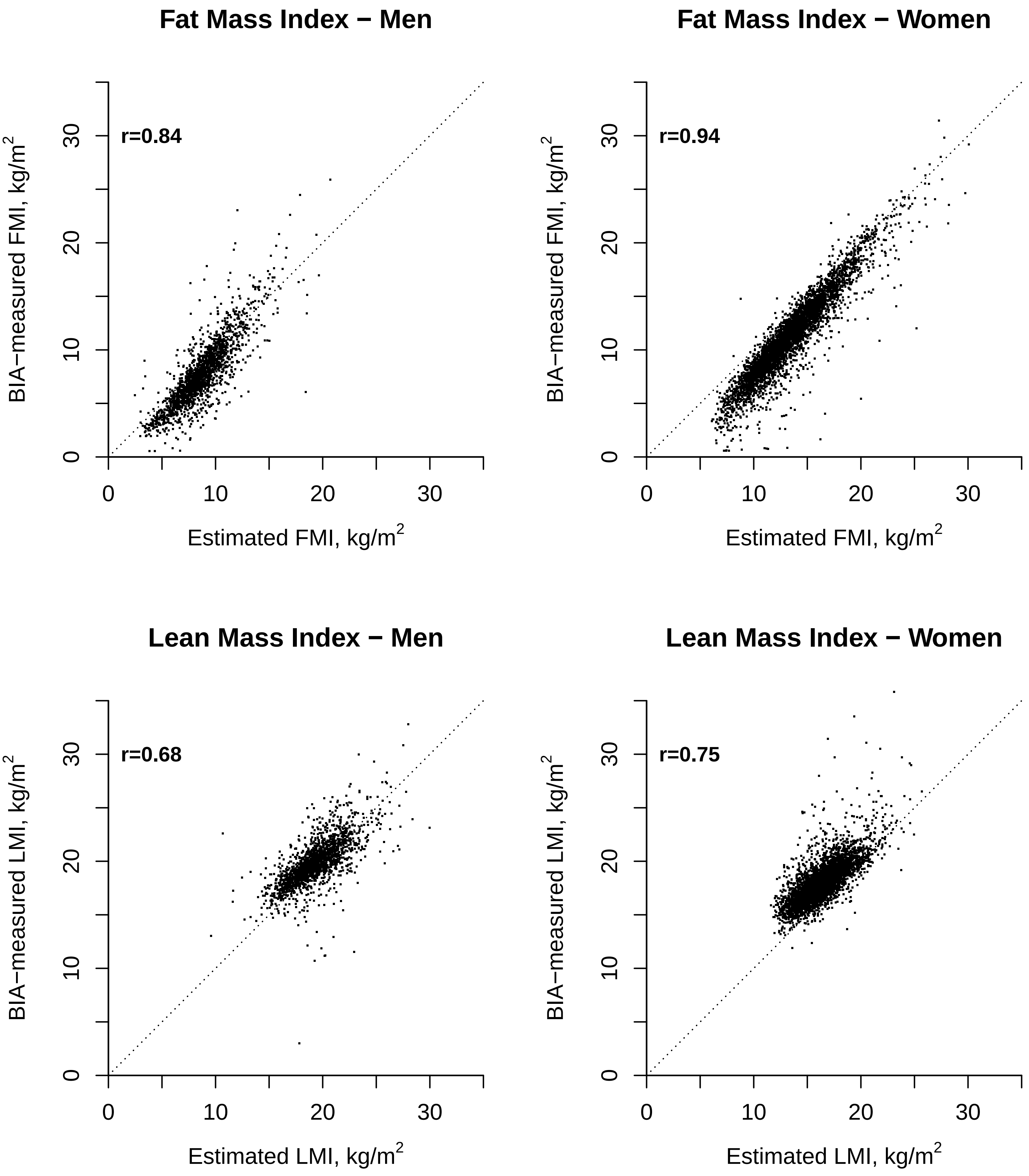

BIA=Bio−electrical Impedance Analysis; r=partial (age−adjusted) Pearson correlation coefficient
Data plotted for 6182 participants who satisfied the criteria for inclusion in the main analyses (see footnote to Table 1), who were aged <90 years at resurvey with both BIA−measured body composition and physical measurements (with BMI in the range 18.5−60 kg/m2) recorded. Estimated values of FMI and LMI calculated using NHANES equations (see footnote to Table 1).

Webfigure 2: Predicted body composition by age and sex in those aged 35−74 and without diabetes or chronic disease at recruitment

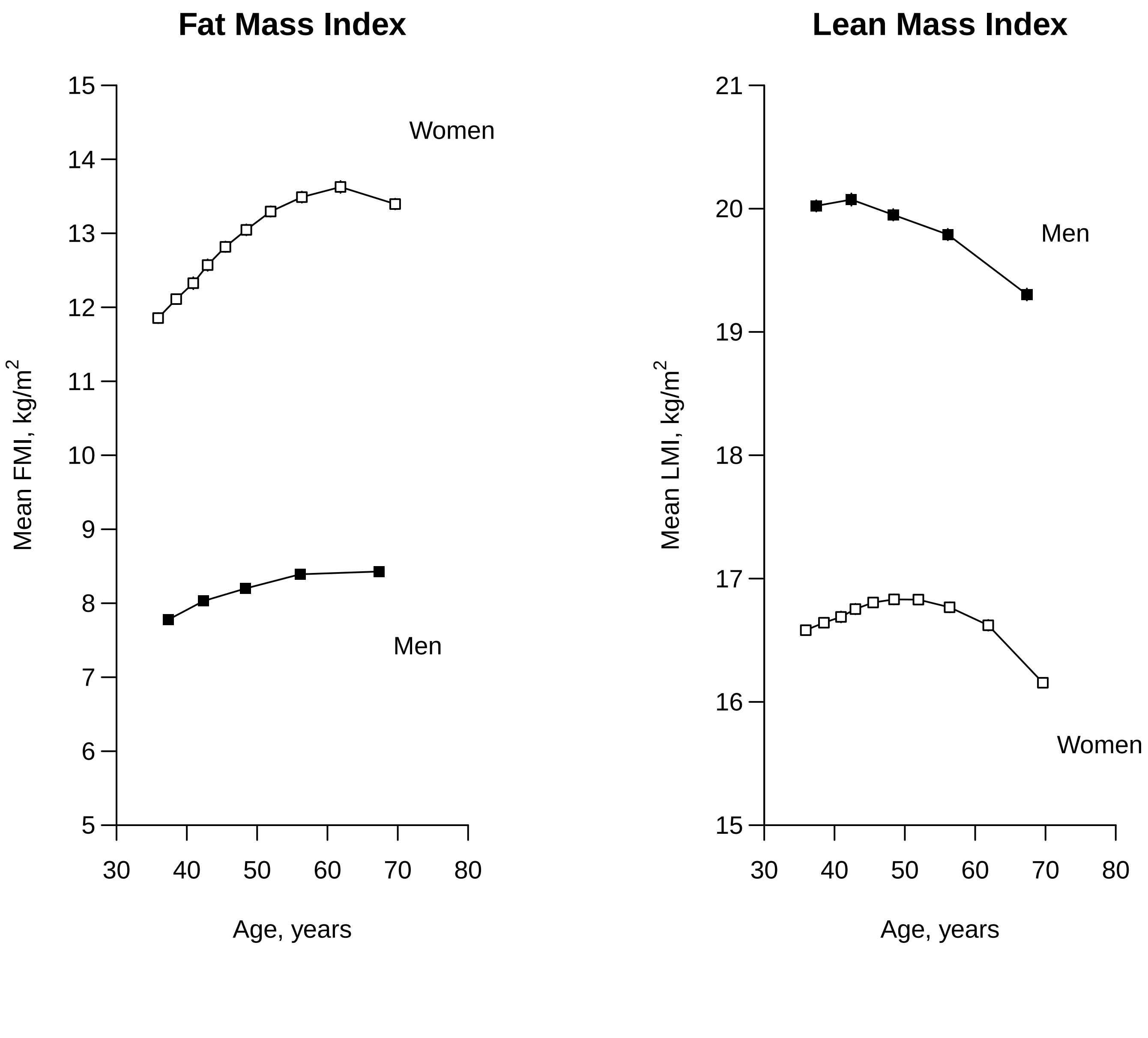

Predicted body composition by age and sex shown for five equally−sized groups of men and ten equally−sized groups of women. Vertical lines representing 95% confidence intervals have been drawn, but are largely contained within the height of each plotting symbol.

Webfigure 3: Relevance of FMI and LMI to IHD mortality, in men and women, at ages 40−74 years

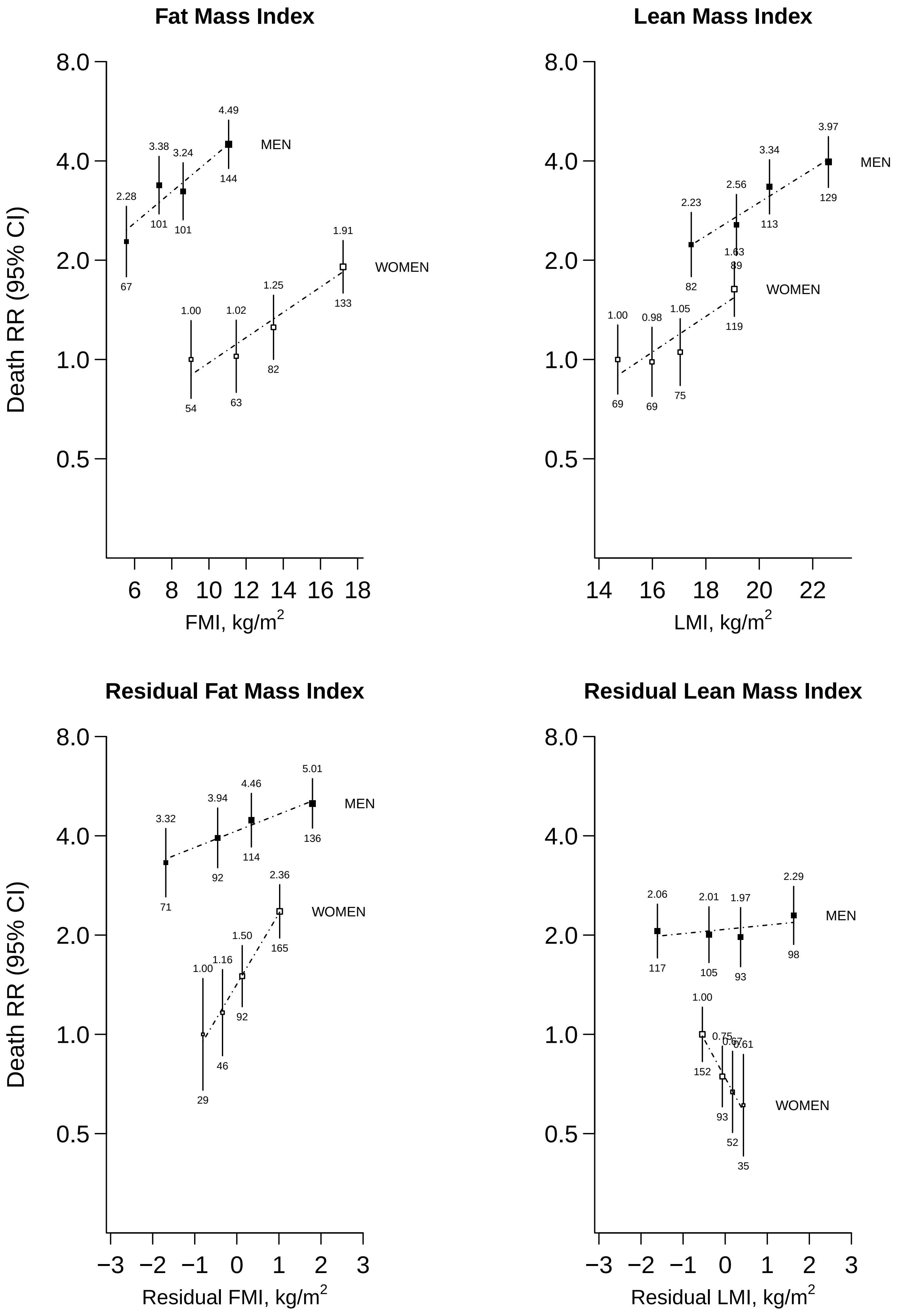

Analyses as for Figure 2, but for IHD mortality (see Webtable 1 and Table 2).

Webfigure 4: Relevance of FMI and LMI to stroke mortality, in men and women, at ages 40−74 years

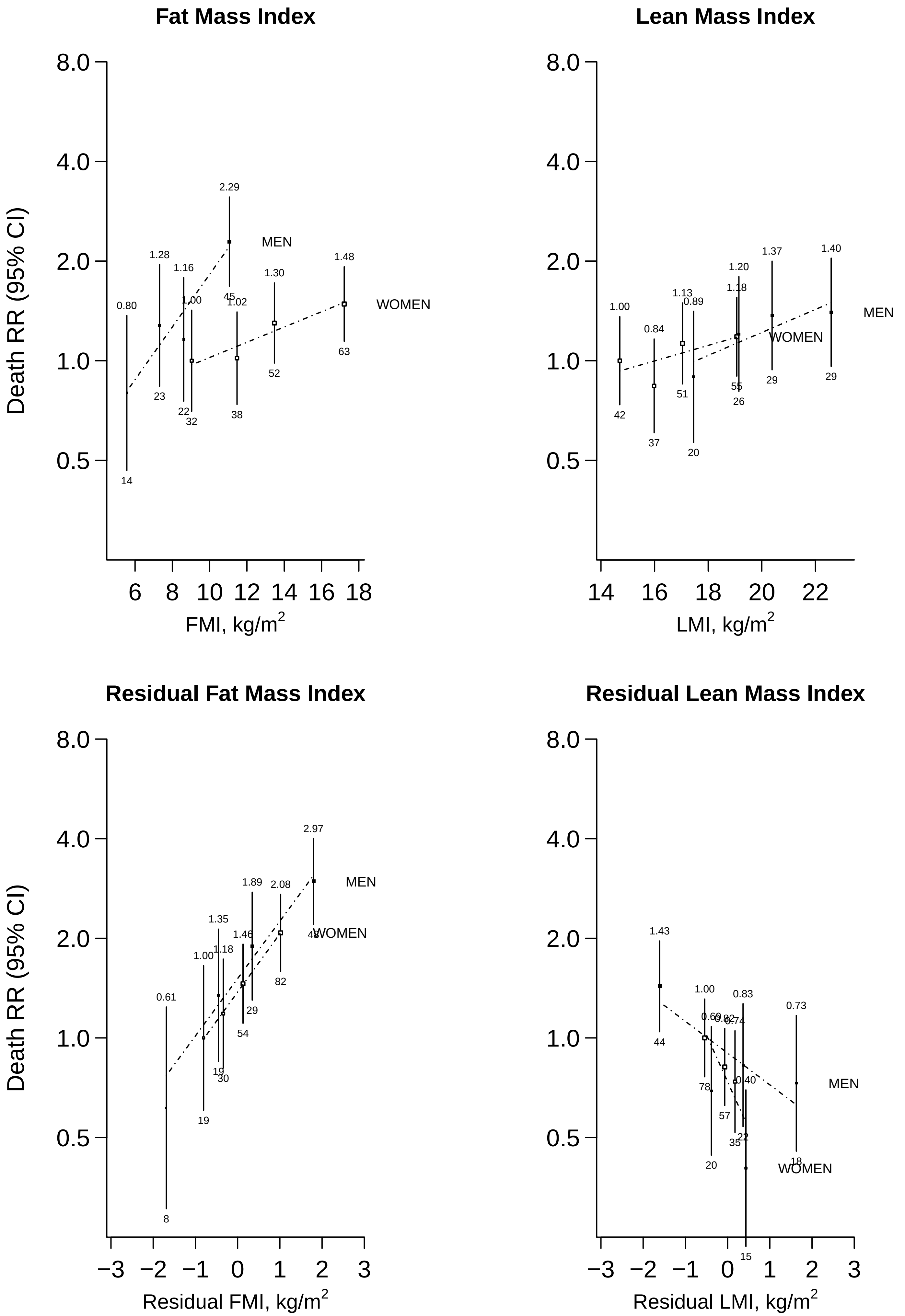

Analyses as for Figure 2, but for stroke mortality (see Webtable 1 and Table 2).

Webfigure 5: Relevance of FMI and LMI to other vascular mortality, in men and women, at ages 40−74 years

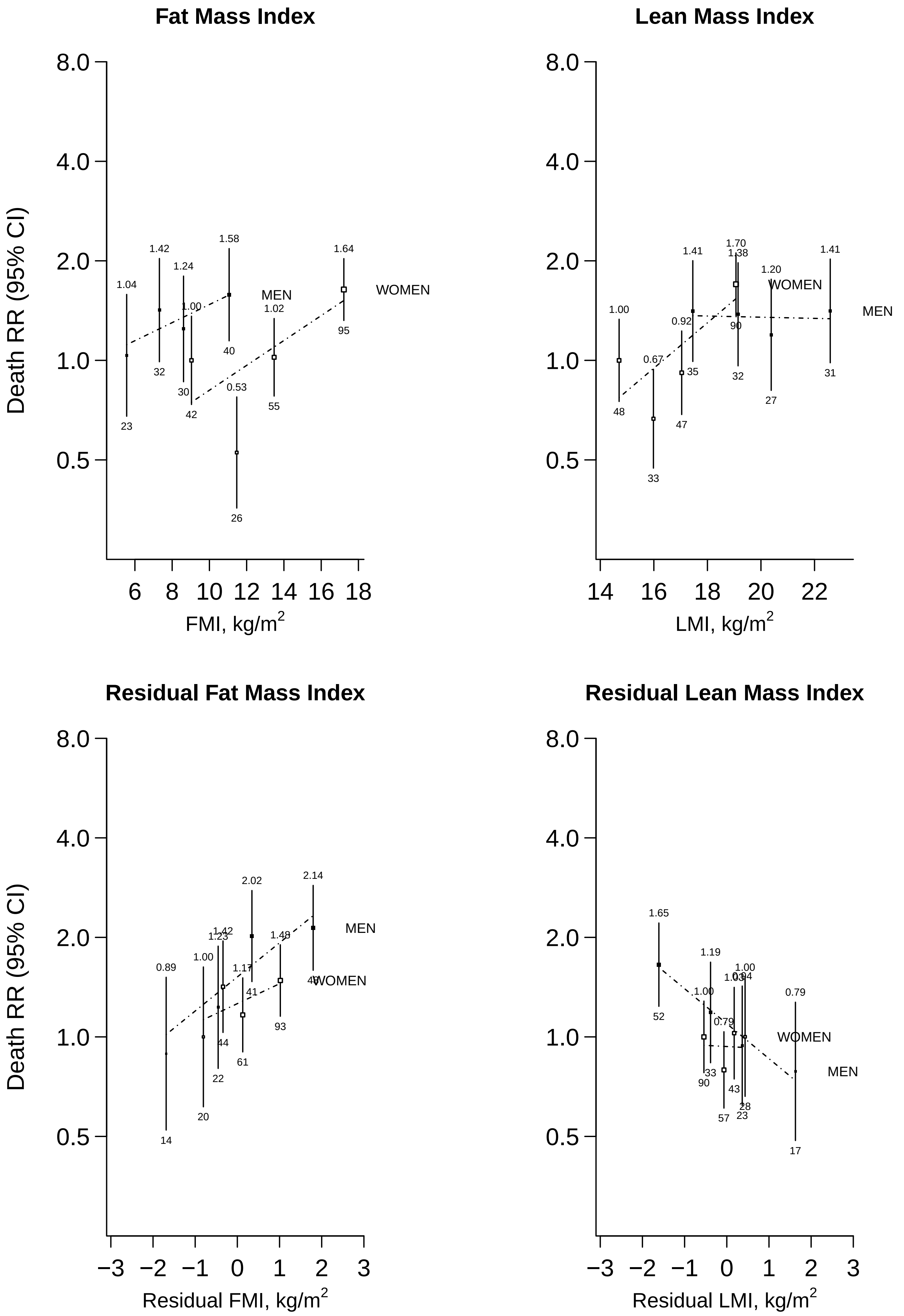

Analyses as for Figure 2, but for other vascular mortality (see Webtable 1 and Table 2).

Webfigure 6: Relevance of FMI and LMI to any vascular mortality, in men and women, at ages 40−74 years

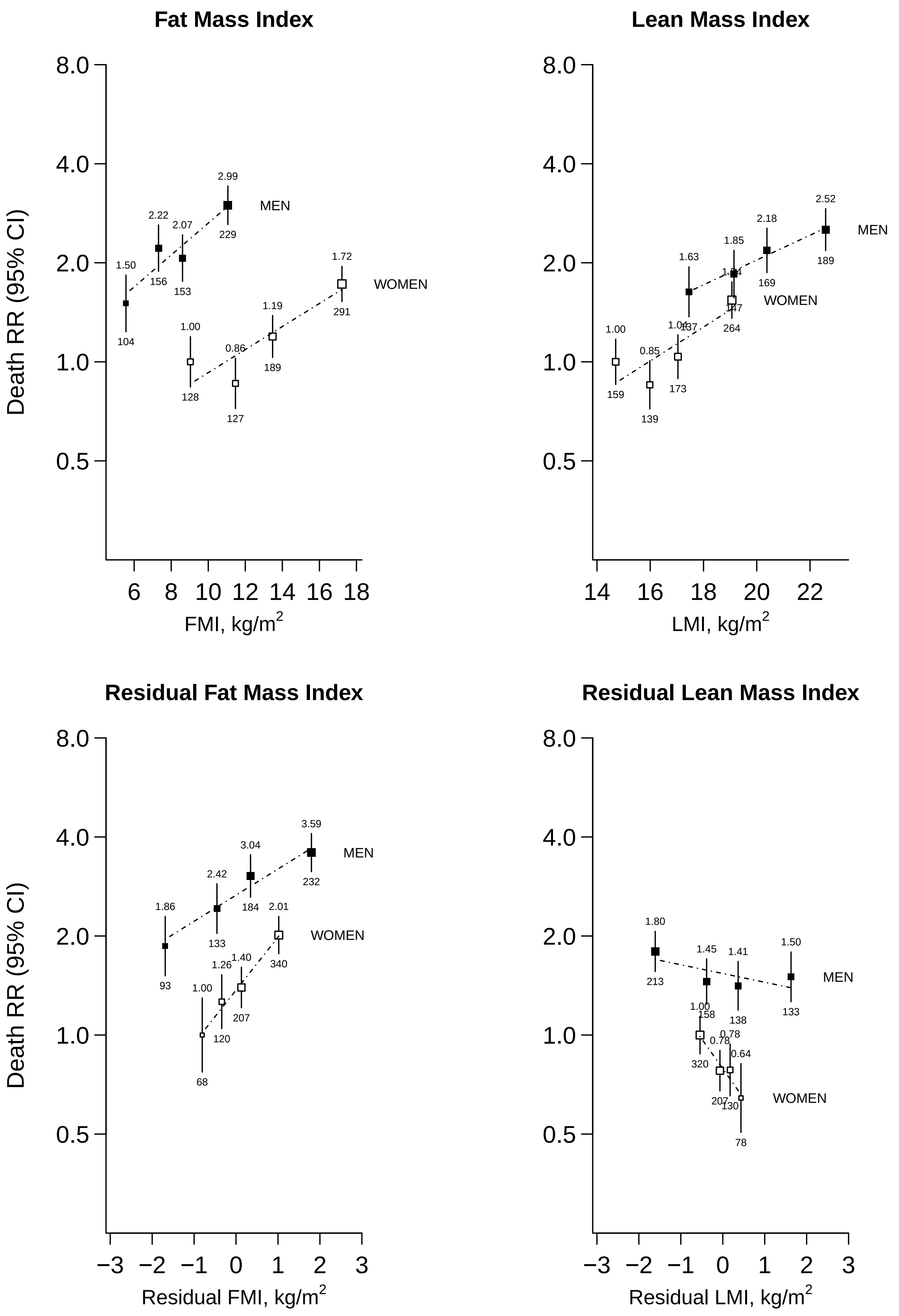

Analyses as for Figure 2, but for any vascular mortality (see Webtable 1 and Table 2).

Webfigure 7: Relevance of FMI and LMI to renal/acute diabetic mortality, in men and women, at ages 40−74 years

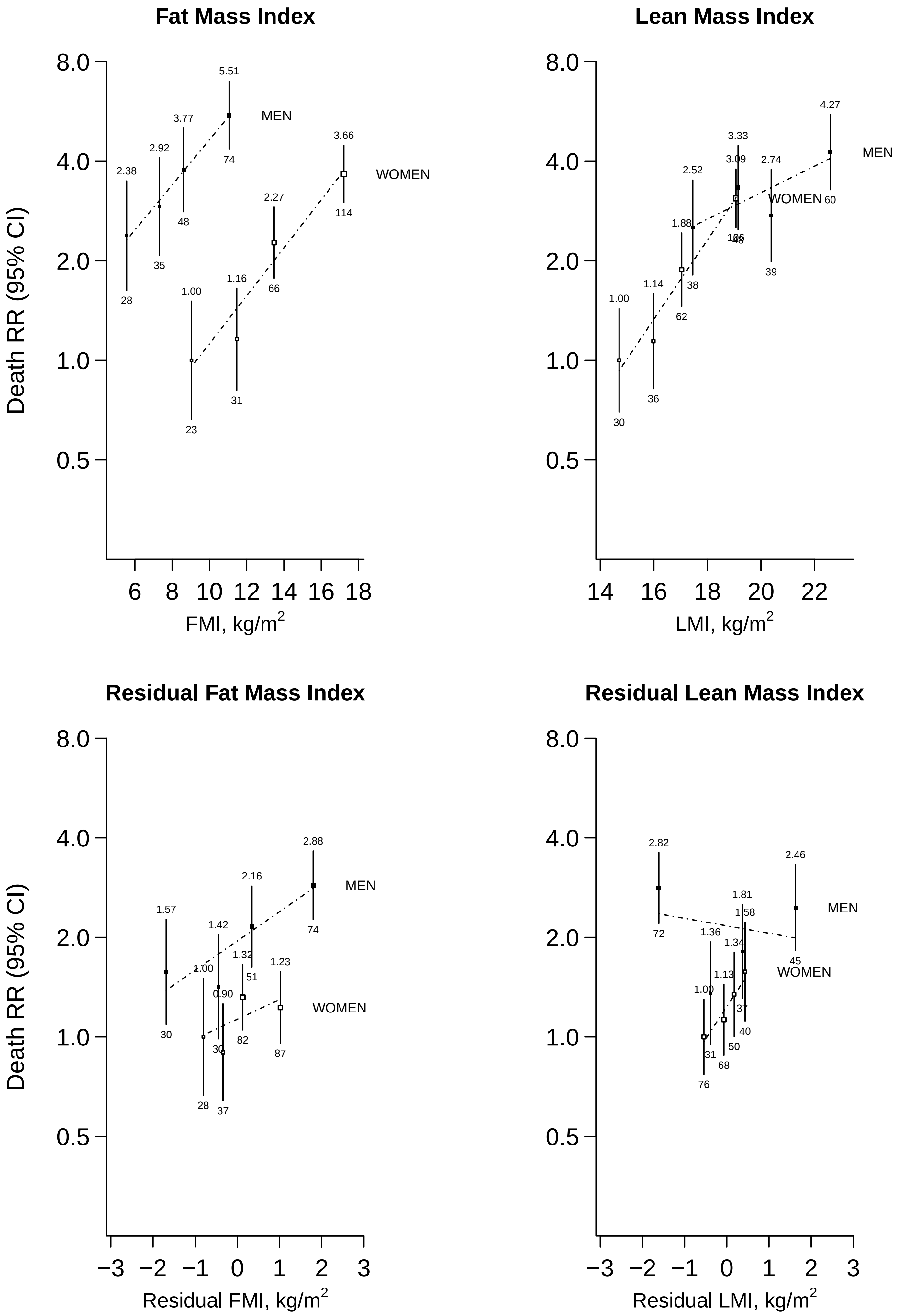

Analyses as for Figure 2, but for renal/acute diabetic mortality (see Webtable 1 and Table 2).

Webfigure 8: Relevance of FMI and LMI to hepatobilary mortality, in men and women, at ages 40−74 years

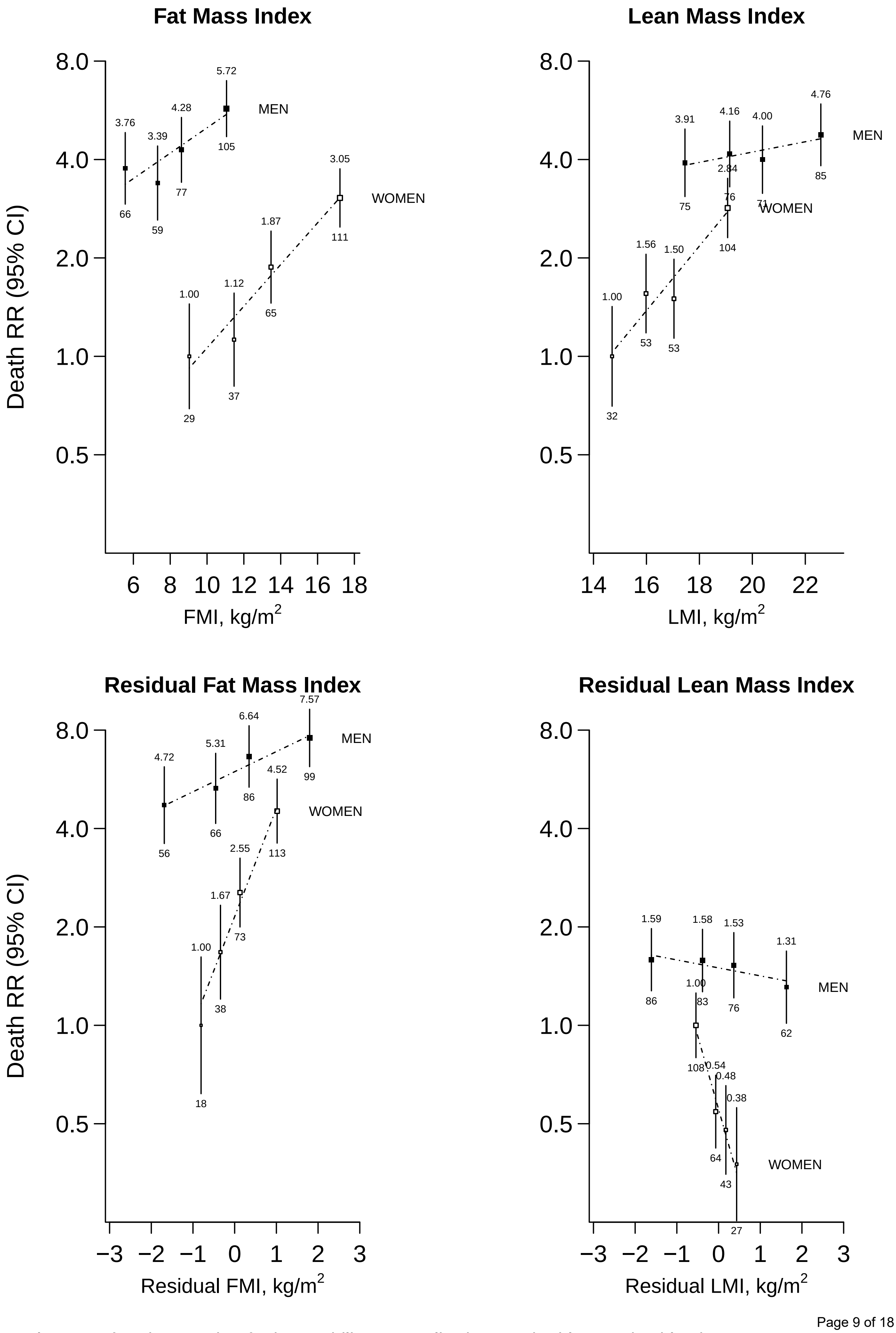

Analyses as for Figure 2, but for hepatobiliary mortality (see Webtable 1 and Table 2).

Webfigure 9: Relevance of FMI and LMI to any metabolic mortality, in men and women, at ages 40−74 years

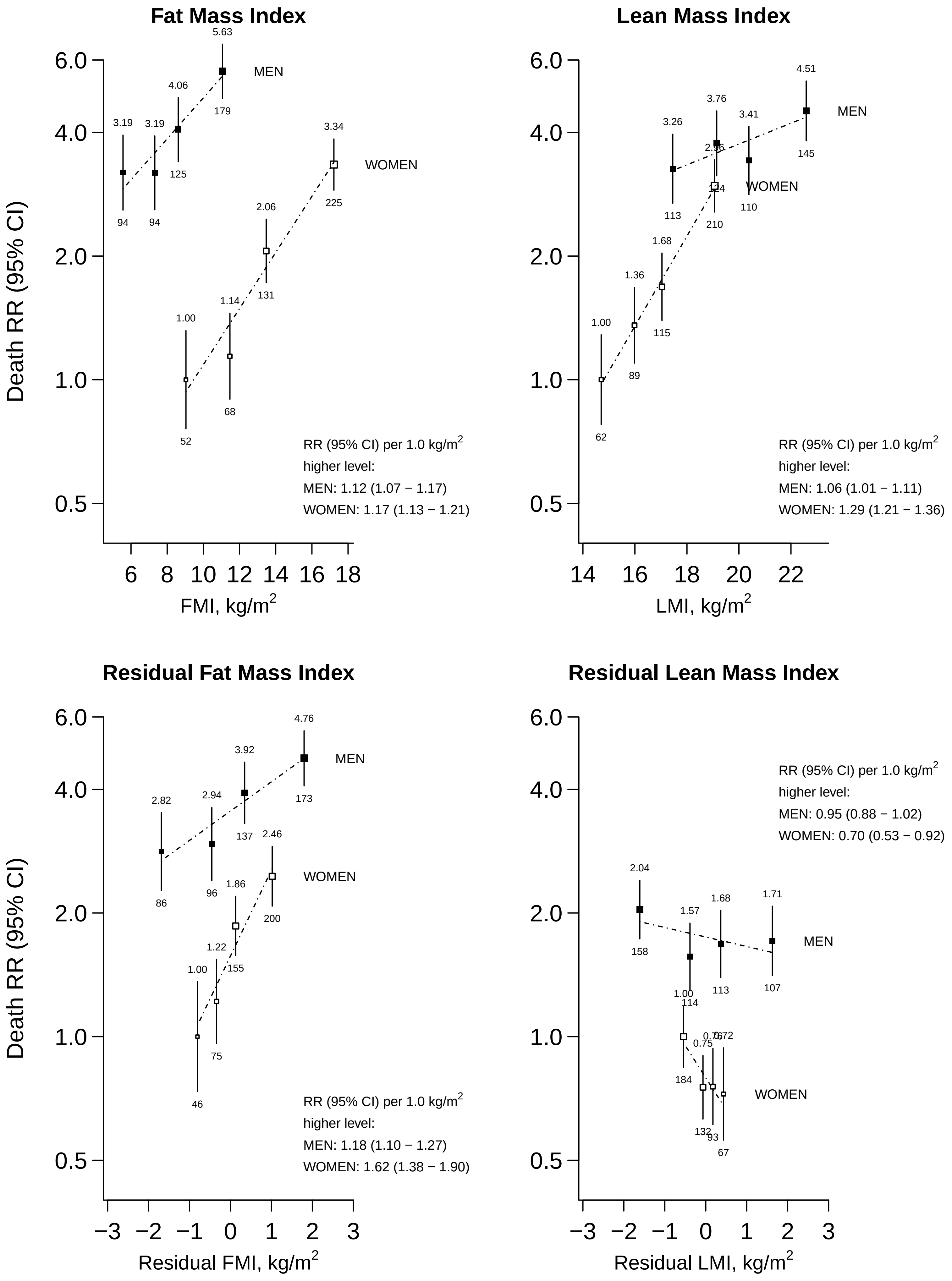

Analyses as for Figure 2, but for any metabolic mortality (see Webtable 1 and Table 2).

Webfigure 10a: Relevance of predicted body composition to vascular−metabolic mortality in men, by levels of confounders

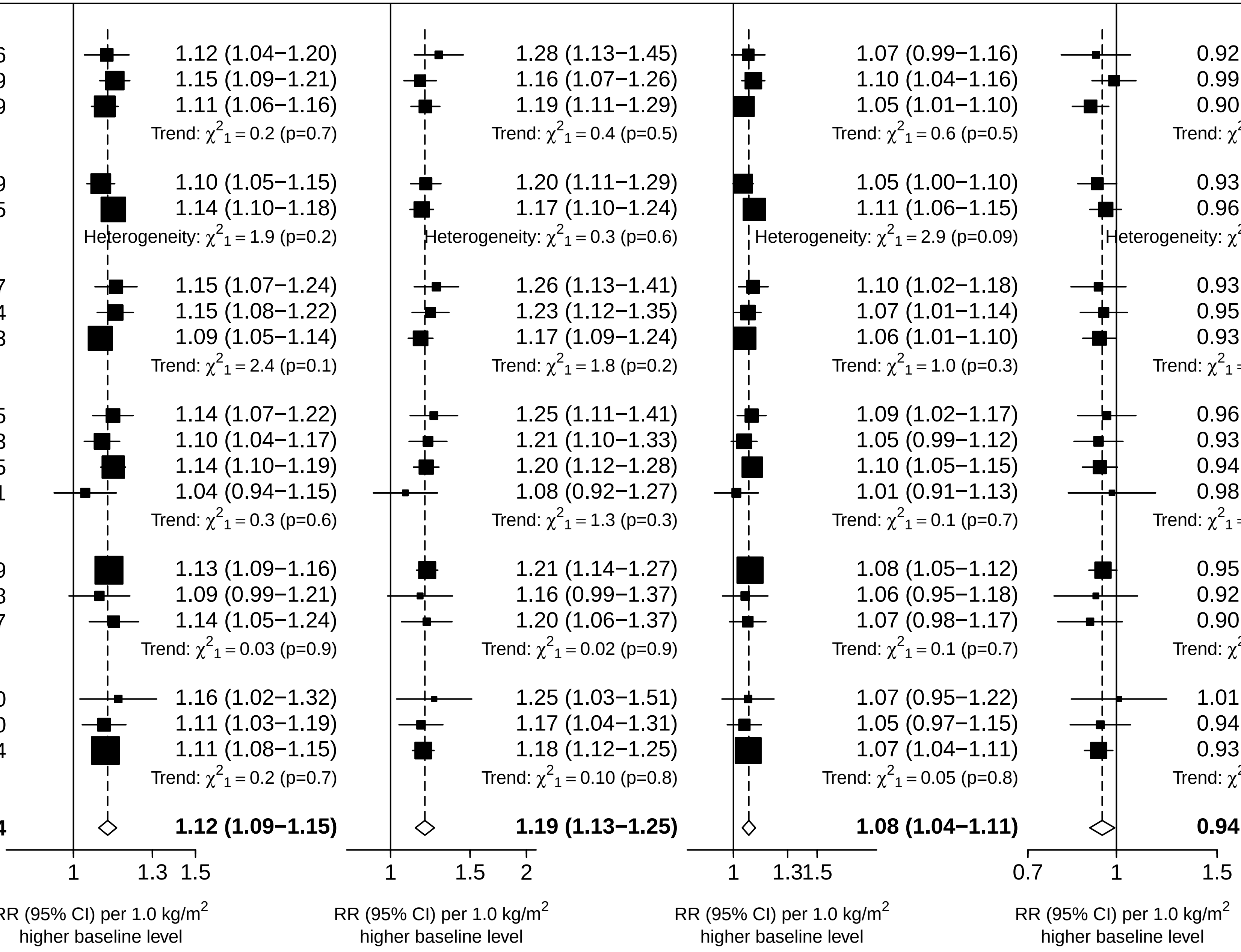

Variable	Number of deaths	FMI Death RR, 95% CI	Residual FMI Death RR, 95% CI	LMI Death RR, 95% CI	Residual LMI Death RR, 95% CI
**Age at risk**					
40−54	206	1.12 (1.04−1.20)	1.28 (1.13−1.45)	1.07 (0.99−1.16)	0.92 (0.80−1.06)
55−64	379	1.15 (1.09−1.21)	1.16 (1.07−1.26)	1.10 (1.04−1.16)	0.99 (0.91−1.08)
65−74	549	1.11 (1.06−1.16)	1.19 (1.11−1.29)	1.05 (1.01−1.10)	0.90 (0.84−0.97)
		Trend: $\chi^2_1 = 0.2$ (p=0.7)	Trend: $\chi^2_1 = 0.4$ (p=0.5)	Trend: $\chi^2_1 = 0.6$ (p=0.5)	Trend: $\chi^2_1 = 0.7$ (p=0.4)
**District**					
Coyoácan	479	1.10 (1.05−1.15)	1.20 (1.11−1.29)	1.05 (1.00−1.10)	0.93 (0.86−1.00)
Iztapalapa	655	1.14 (1.10−1.18)	1.17 (1.10−1.24)	1.11 (1.06−1.15)	0.96 (0.90−1.02)
		Heterogeneity: $\chi^2_1 = 1.9$ (p=0.2)	Heterogeneity: $\chi^2_1 = 0.3$ (p=0.6)	Heterogeneity: $\chi^2_1 = 2.9$ (p=0.09)	Heterogeneity: $\chi^2_1 = 0.4$ (p=0.5)
**Smoking**					
Never	197	1.15 (1.07−1.24)	1.26 (1.13−1.41)	1.10 (1.02−1.18)	0.93 (0.83−1.04)
Former	284	1.15 (1.08−1.22)	1.23 (1.12−1.35)	1.07 (1.01−1.14)	0.95 (0.86−1.04)
Current	653	1.09 (1.05−1.14)	1.17 (1.09−1.24)	1.06 (1.01−1.10)	0.93 (0.87−1.00)
		Trend: $\chi^2_1 = 2.4$ (p=0.1)	Trend: $\chi^2_1 = 1.8$ (p=0.2)	Trend: $\chi^2_1 = 1.0$ (p=0.3)	Trend: $\chi^2_1 = 0.00$ (p=0.97)
**Education**					
University	215	1.14 (1.07−1.22)	1.25 (1.11−1.41)	1.09 (1.02−1.17)	0.96 (0.86−1.08)
High school	293	1.10 (1.04−1.17)	1.21 (1.10−1.33)	1.05 (0.99−1.12)	0.93 (0.84−1.03)
Elementary	545	1.14 (1.10−1.19)	1.20 (1.12−1.28)	1.10 (1.05−1.15)	0.94 (0.87−1.00)
Other	81	1.04 (0.94−1.15)	1.08 (0.92−1.27)	1.01 (0.91−1.13)	0.98 (0.82−1.17)
		Trend: $\chi^2_1 = 0.3$ (p=0.6)	Trend: $\chi^2_1 = 1.3$ (p=0.3)	Trend: $\chi^2_1 = 0.1$ (p=0.7)	Trend: $\chi^2_1 = 0.00$ (p=0.97)
**Physical activity**					
None	849	1.13 (1.09−1.16)	1.21 (1.14−1.27)	1.08 (1.05−1.12)	0.95 (0.90−1.00)
Up to twice weekly	118	1.09 (0.99−1.21)	1.16 (0.99−1.37)	1.06 (0.95−1.18)	0.92 (0.78−1.09)
At least 3 times weekly	167	1.14 (1.05−1.24)	1.20 (1.06−1.37)	1.07 (0.98−1.17)	0.90 (0.79−1.02)
		Trend: $\chi^2_1 = 0.03$ (p=0.9)	Trend: $\chi^2_1 = 0.02$ (p=0.9)	Trend: $\chi^2_1 = 0.1$ (p=0.7)	Trend: $\chi^2_1 = 0.6$ (p=0.5)
**Alcohol consumption**					
None	60	1.16 (1.02−1.32)	1.25 (1.03−1.51)	1.07 (0.95−1.22)	1.01 (0.83−1.22)
Former	180	1.11 (1.03−1.19)	1.17 (1.04−1.31)	1.05 (0.97−1.15)	0.94 (0.83−1.06)
Current	894	1.11 (1.08−1.15)	1.18 (1.12−1.25)	1.07 (1.04−1.11)	0.93 (0.88−0.99)
		Trend: $\chi^2_1 = 0.2$ (p=0.7)	Trend: $\chi^2_1 = 0.10$ (p=0.8)	Trend: $\chi^2_1 = 0.05$ (p=0.8)	Trend: $\chi^2_1 = 0.5$ (p=0.5)
**Overall**	**1134**	**1.12 (1.09−1.15)**	**1.19 (1.13−1.25)**	**1.08 (1.04−1.11)**	**0.94 (0.90−0.99)**

Exclusions, conventions and analyses as for Figure 2 and Table 2, with the exception that RRs are now estimated separately by levels of each confounder shown (with mutual adjustment for the other confounders).

Webfigure 10b: Relevance of predicted body composition to vascular−metabolic mortality in women, by levels of confounders

Variable	Number of deaths	FMI: Death RR, 95% CI	Residual FMI: Death RR, 95% CI	LMI: Death RR, 95% CI	Residual LMI: Death RR, 95% CI
**Age at risk**					
40−54	171	1.11 (1.06−1.17)	2.03 (1.32−3.15)	1.15 (1.04−1.27)	0.50 (0.22−1.13)
55−64	404	1.14 (1.10−1.18)	1.60 (1.32−1.94)	1.25 (1.18−1.32)	0.67 (0.47−0.96)
65−74	636	1.09 (1.06−1.13)	1.27 (1.06−1.53)	1.15 (1.10−1.21)	1.04 (0.77−1.42)
		Trend: $\chi^2_1 = 1.5$ (p=0.2)	Trend: $\chi^2_1 = 5.1$ (p=0.02)	Trend: $\chi^2_1 = 0.7$ (p=0.4)	Trend: $\chi^2_1 = 4.9$ (p=0.03)
**District**					
Coyoácan	414	1.14 (1.10−1.17)	1.78 (1.52−2.08)	1.21 (1.14−1.28)	0.51 (0.38−0.68)
Iztapalapa	797	1.10 (1.08−1.13)	1.37 (1.22−1.54)	1.18 (1.13−1.23)	0.77 (0.62−0.94)
		Heterogeneity: $\chi^2_1 = 2.0$ (p=0.2)	Heterogeneity: $\chi^2_1 = 7.2$ (p=0.007)	Heterogeneity: $\chi^2_1 = 0.4$ (p=0.5)	Heterogeneity: $\chi^2_1 = 4.9$ (p=0.03)
**Smoking**					
Never	758	1.13 (1.11−1.16)	1.41 (1.25−1.58)	1.22 (1.17−1.27)	0.79 (0.64−0.97)
Former	166	1.09 (1.03−1.14)	1.44 (1.13−1.82)	1.15 (1.06−1.26)	0.57 (0.38−0.87)
Current	287	1.08 (1.04−1.13)	1.79 (1.44−2.22)	1.13 (1.05−1.21)	0.55 (0.37−0.81)
		Trend: $\chi^2_1 = 4.3$ (p=0.04)	Trend: $\chi^2_1 = 3.1$ (p=0.08)	Trend: $\chi^2_1 = 3.7$ (p=0.05)	Trend: $\chi^2_1 = 3.2$ (p=0.07)
**Education**					
University	79	1.19 (1.10−1.28)	1.51 (0.95−2.38)	1.40 (1.21−1.62)	0.90 (0.38−2.12)
High school	265	1.09 (1.05−1.13)	2.14 (1.68−2.73)	1.13 (1.05−1.21)	0.42 (0.28−0.65)
Elementary	702	1.12 (1.09−1.14)	1.47 (1.29−1.67)	1.19 (1.14−1.25)	0.69 (0.55−0.86)
Other	165	1.10 (1.04−1.15)	1.02 (0.82−1.27)	1.18 (1.07−1.29)	1.30 (0.88−1.92)
		Trend: $\chi^2_1 = 0.5$ (p=0.5)	Trend: $\chi^2_1 = 13.7$ (p<0.001)	Trend: $\chi^2_1 = 0.5$ (p=0.5)	Trend: $\chi^2_1 = 8.5$ (p=0.004)
**Physical activity**					
None	1050	1.11 (1.09−1.14)	1.53 (1.38−1.70)	1.17 (1.13−1.21)	0.64 (0.53−0.77)
Up to twice weekly	43	1.22 (1.09−1.35)	1.43 (0.92−2.22)	1.42 (1.16−1.73)	0.82 (0.38−1.81)
At least 3 times weekly	118	1.11 (1.04−1.18)	1.32 (0.99−1.76)	1.22 (1.08−1.36)	0.92 (0.53−1.58)
		Trend: $\chi^2_1 = 0.1$ (p=0.7)	Trend: $\chi^2_1 = 0.9$ (p=0.3)	Trend: $\chi^2_1 = 1.1$ (p=0.3)	Trend: $\chi^2_1 = 1.8$ (p=0.2)
**Alcohol consumption**					
None	339	1.09 (1.05−1.12)	1.36 (1.15−1.60)	1.14 (1.07−1.21)	0.68 (0.50−0.91)
Former	175	1.11 (1.06−1.16)	1.38 (1.09−1.74)	1.17 (1.07−1.28)	0.72 (0.48−1.08)
Current	697	1.13 (1.10−1.15)	1.51 (1.33−1.71)	1.22 (1.16−1.28)	0.64 (0.51−0.81)
		Trend: $\chi^2_1 = 2.8$ (p=0.09)	Trend: $\chi^2_1 = 1.0$ (p=0.3)	Trend: $\chi^2_1 = 3.2$ (p=0.07)	Trend: $\chi^2_1 = 0.10$ (p=0.8)
**Overall**	**1211**	**1.12 (1.09−1.14)**	**1.52 (1.38−1.68)**	**1.19 (1.14−1.23)**	**0.67 (0.56−0.80)**

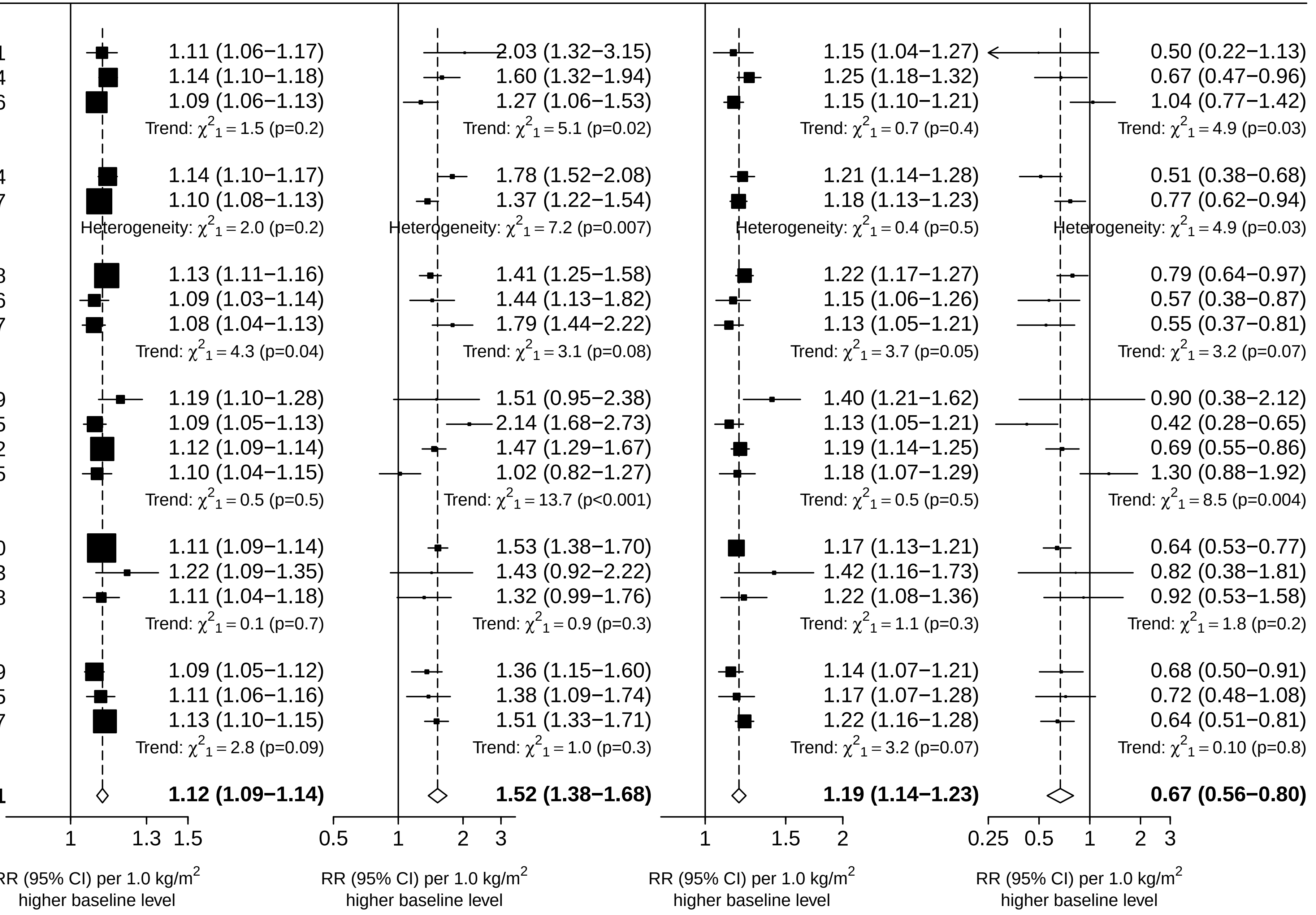

Exclusions, conventions and analyses as for Figure 2 and Table 2, with the exception that RRs are now estimated separately by levels of each confounder shown (with mutual adjustment for the other confounders).

Webtable 1: Numbers of deaths at ages 40-74 by underlying cause (ICD-10 code)

Underlying cause of death	ICD-10 codes (and number of deaths)
IHD (n=745)	I200 (1), I209 (2), I210 (1), I211 (4), I219 (690), I221 (1), I249 (5), I251 (10), I258 (3), I259 (28)
Stroke (n=289)	F019 (2), I600 (2), I609 (38), I610 (1), I613 (1), I615 (1), I619 (85), I620 (5), I629 (3), I633 (1), I634 (8), I639 (20), I64X (44), I671 (6), I673 (1), I678 (32), I679 (27), I690 (2), I693 (1), I694 (2), I698 (7)
Other vascular (n=343)	E115 (3), E145 (2), I018 (1), I050 (1), I051 (2), I059 (7), I070 (1), I071 (2), I080 (2), I081 (1), I091 (1), I099 (11), I110 (50), I119 (8), I260 (1), I269 (37), I270 (5), I272 (1), I279 (1), I301 (1), I330 (3), I340 (3), I350 (7), I351 (1), I358 (1), I38X (3), I420 (6), I429 (1), I442 (2), I471 (2), I472 (1), I480 (1), I489 (3), I48X (3), I490 (4), I499 (1), I500 (31), I501 (4), I509 (31), I518 (3), I519 (3), I710 (2), I712 (1), I713 (3), I714 (1), I718 (1), I719 (1), I729 (1), I739 (2), I740 (1), I741 (1), I771 (1), I776 (1), I802 (3), I822 (1), I828 (1), I829 (1), I872 (4), I879 (1), I890 (1), I99X (1), K550 (32), K559 (7), K761 (1), Q210 (1), Q231 (1), Q238 (1), Q248 (1), R570 (18)
Renal (n=321)	E102 (1), E112 (56), E142 (16), I120 (43), I129 (2), I131 (1), I132 (6), N002 (1), N039 (4), N059 (6), N12X (1), N151 (6), N179 (31), N180 (5), N185 (2), N189 (52), N19X (9), N200 (4), N201 (1), N289 (1), N300 (1), N309 (1), N390 (71)
Acute diabetic crisis (n=98)	E110 (31), E111 (36), E140 (13), E141 (17), E162 (1)
Hepatobiliary (n=549)	B169 (1), B171 (9), B181 (1), B182 (6), B189 (1), B190 (2), B199 (2), D136 (1), I850 (8), I859 (3), K701 (14), K702 (1), K703 (75), K704 (16), K709 (10), K716 (1), K720 (7), K721 (43), K729 (117), K745 (2), K746 (133), K750 (7), K754 (2), K759 (1), K760 (1), K764 (1), K766 (3), K767 (5), K768 (1), K769 (5), K800 (3), K801 (5), K802 (1), K803 (3), K805 (1), K810 (4), K811 (3), K819 (3), K821 (1), K829 (3), K830 (9), K831 (2), K851 (1), K852 (3), K858 (1), K859 (22), K85X (2), K861 (2), Q447 (1)

Webtable 2a: Characteristics of 113 025 participants aged 35-74 at recruitment, by sex and fat mass index

	Men					Women				
	FMI group* (participants)					FMI group* (participants)				
	I (9 153)	II (9 153)	III (9 153)	IV (9 153)	Total (36 612)	I (19 103)	II (19 104)	III (19 103)	IV (19 103)	Total (76 413)
Age, years	48 (11)	49 (11)	50 (11)	51 (11)	50 (11)	46 (10)	48 (10)	50 (10)	51 (10)	49 (10)
**Socio-economic status and lifestyle behaviours**										
Resident of Coyoacán	4637 (51%)	4265 (47%)	3989 (44%)	3620 (40%)	16 511 (45%)	9055 (47%)	7853 (41%)	6969 (36%)	6457 (34%)	30 334 (40%)
University/college educated	2848 (31%)	2728 (30%)	2380 (26%)	2017 (22%)	9973 (27%)	4344 (23%)	2730 (14%)	1991 (10%)	1532 (8%)	10 597 (14%)
Current smoker	5265 (58%)	4746 (52%)	4585 (50%)	4460 (49%)	19 056 (52%)	5626 (29%)	4971 (26%)	4440 (23%)	4165 (22%)	19 202 (25%)
Current drinker	7318 (80%)	7351 (80%)	7301 (80%)	7215 (79%)	29 185 (80%)	12 773 (67%)	12 641 (66%)	12 328 (65%)	12 018 (63%)	49 760 (65%)
Any regular leisure-time physical activity	3278 (36%)	3208 (35%)	2748 (30%)	2249 (25%)	11 483 (31%)	4676 (24%)	4079 (21%)	3287 (17%)	2641 (14%)	14 683 (19%)
**Biological measurements**										
SBP, mmHg	123 (14)	126 (14)	128 (14)	132 (15)	127 (15)	119 (15)	123 (15)	126 (16)	130 (16)	124 (16)
DBP, mmHg	81 (9)	83 (9)	85 (9)	87 (10)	84 (10)	78 (10)	81 (10)	83 (10)	85 (10)	82 (10)
Glycated haemoglobin, mean (SD), %	5.3 (0.3)	5.4 (0.3)	5.5 (0.4)	5.6 (0.4)	5.4 (0.4)	5.3 (0.3)	5.4 (0.4)	5.5 (0.4)	5.6 (0.4)	5.5 (0.4)
**Long term medication use**										
Any anti-hypertensive	306 (3%)	536 (6%)	754 (8%)	1156 (13%)	2752 (8%)	1203 (6%)	1930 (10%)	2659 (14%)	3887 (20%)	9679 (13%)
Any anti-thrombotic	132 (1%)	151 (2%)	207 (2%)	241 (3%)	731 (2%)	416 (2%)	409 (2%)	506 (3%)	608 (3%)	1939 (3%)
Any lipid lowering	29 (<0.5%)	37 (<0.5%)	43 (<0.5%)	41 (<0.5%)	150 (<0.5%)	74 (<0.5%)	87 (<0.5%)	82 (<0.5%)	69 (<0.5%)	312 (<0.5%)
**Physical measurements**										
Height, cm	165 (7)	165 (7)	165 (7)	164 (7)	165 (7)	154 (6)	152 (6)	151 (6)	151 (6)	152 (6)
Weight, kg	65 (7)	73 (7)	79 (8)	89 (12)	76 (13)	56 (6)	64 (6)	70 (6)	83 (11)	68 (12)
BMI, kg/m²	23.6 (1.9)	26.5 (1.4)	28.8 (1.5)	33.0 (3.2)	28.0 (4.1)	23.8 (1.7)	27.5 (0.9)	30.5 (1.1)	36.2 (3.7)	29.5 (5.0)
Waist circumference, cm	85 (5)	93 (3)	98 (4)	108 (8)	96 (10)	81 (7)	88 (6)	95 (6)	106 (9)	92 (12)
Hip circumference, cm	94 (5)	99 (5)	103 (5)	109 (8)	101 (8)	96 (6)	102 (6)	108 (6)	119 (10)	106 (11)
Waist-hip ratio	0.90 (0.05)	0.94 (0.04)	0.96 (0.04)	0.99 (0.06)	0.95 (0.06)	0.84 (0.06)	0.87 (0.06)	0.88 (0.06)	0.89 (0.06)	0.87 (0.06)
Fat mass, kg *	15 (3)	20 (2)	24 (2)	30 (5)	22 (6)	21 (3)	27 (2)	31 (3)	39 (6)	30 (8)
Lean mass, kg **	49 (5)	53 (5)	55 (6)	59 (8)	54 (7)	35 (4)	37 (4)	39 (4)	43 (5)	39 (5)
Fat mass index, kg/m² ***	5.6 (0.9)	7.3 (0.4)	8.6 (0.4)	11.1 (1.7)	8.2 (2.2)	9.1 (1.1)	11.5 (0.6)	13.5 (0.6)	17.2 (2.4)	12.8 (3.3)
Lean mass index, kg/m² $	18.0 (1.4)	19.2 (1.3)	20.2 (1.3)	21.9 (2.0)	19.8 (2.1)	14.7 (0.7)	16.0 (0.5)	17.0 (0.6)	19.0 (1.4)	16.7 (1.8)

Conventions and exclusions as per Table 1. * Groupings of fat mass index are sex-specific quartiles of the baseline distribution.

Webtable 2b: Characteristics of 113 025 participants aged 35-74 at recruitment, by sex and lean mass index

	Men<br>LMI group* (participants)					Women<br>LMI group* (participants)				
	**I (9 153)**	**II (9 154)**	**III (9 152)**	**IV (9 153)**	**Total (36 612)**	**I (19 104)**	**II (19 102)**	**III (19 105)**	**IV (19 102)**	**Total (76 413)**
Age, years	52 (11)	50 (11)	49 (10)	48 (10)	50 (11)	50 (11)	49 (10)	48 (10)	48 (10)	49 (10)
**Socio-economic status and lifestyle behaviours**										
Resident of Coyoacán	4556 (50%)	4344 (47%)	3974 (43%)	3637 (40%)	16 511 (45%)	8921 (47%)	7928 (42%)	7034 (37%)	6451 (34%)	30 334 (40%)
University/college educated	2505 (27%)	2599 (28%)	2560 (28%)	2309 (25%)	9973 (27%)	3705 (19%)	2770 (15%)	2289 (12%)	1833 (10%)	10 597 (14%)
Current smoker	5019 (55%)	4697 (51%)	4664 (51%)	4676 (51%)	19 056 (52%)	5102 (27%)	4883 (26%)	4609 (24%)	4608 (24%)	19 202 (25%)
Current drinker	7204 (79%)	7266 (79%)	7393 (81%)	7322 (80%)	29 185 (80%)	12 331 (65%)	12 643 (66%)	12 519 (66%)	12 267 (64%)	49 760 (65%)
Any regular leisure-time physical activity	2816 (31%)	3000 (33%)	3051 (33%)	2616 (29%)	11 483 (31%)	4545 (24%)	4202 (22%)	3270 (17%)	2666 (14%)	14 683 (19%)
**Biological measurements**										
SBP, mmHg	125 (15)	126 (14)	127 (14)	130 (15)	127 (15)	121 (16)	123 (15)	125 (15)	129 (15)	124 (16)
DBP, mmHg	82 (10)	84 (9)	84 (9)	86 (10)	84 (10)	79 (10)	81 (10)	82 (10)	85 (10)	82 (10)
Glycated haemoglobin, mean (SD), %	5.4 (0.3)	5.4 (0.3)	5.5 (0.4)	5.5 (0.4)	5.4 (0.4)	5.3 (0.4)	5.4 (0.4)	5.5 (0.4)	5.6 (0.4)	5.5 (0.4)
**Long term medication use**										
Any anti-hypertensive	509 (6%)	629 (7%)	699 (8%)	915 (10%)	2752 (8%)	1777 (9%)	2076 (11%)	2454 (13%)	3372 (18%)	9679 (13%)
Any anti-thrombotic	160 (2%)	197 (2%)	183 (2%)	191 (2%)	731 (2%)	479 (3%)	425 (2%)	489 (3%)	546 (3%)	1939 (3%)
Any lipid lowering	30 (<0.5%)	37 (<0.5%)	50 (1%)	33 (<0.5%)	150 (<0.5%)	93 (<0.5%)	85 (<0.5%)	70 (<0.5%)	64 (<0.5%)	312 (<0.5%)
**Physical measurements**										
Height, cm	165 (7)	165 (7)	165 (7)	165 (7)	165 (7)	152 (7)	152 (6)	152 (6)	152 (6)	152 (6)
Weight, kg	65 (7)	72 (7)	78 (7)	90 (11)	76 (13)	56 (6)	64 (5)	70 (5)	83 (10)	68 (12)
BMI, kg/m²	23.6 (1.9)	26.6 (1.5)	28.8 (1.5)	33.0 (3.3)	28.0 (4.1)	23.9 (1.8)	27.5 (1.2)	30.5 (1.2)	36.2 (3.7)	29.5 (5.0)
Waist circumference, cm	89 (8)	93 (7)	97 (8)	104 (11)	96 (10)	82 (8)	89 (7)	94 (7)	104 (10)	92 (12)
Hip circumference, cm	96 (6)	99 (5)	102 (6)	108 (8)	101 (8)	96 (6)	102 (6)	108 (6)	118 (10)	106 (11)
Waist-hip ratio	0.93 (0.06)	0.94 (0.05)	0.95 (0.06)	0.96 (0.06)	0.95 (0.06)	0.85 (0.07)	0.87 (0.06)	0.88 (0.06)	0.88 (0.06)	0.87 (0.06)
Fat mass, kg *	17 (4)	20 (4)	23 (4)	28 (6)	22 (6)	21 (3)	27 (2)	31 (3)	39 (6)	30 (8)
Lean mass, kg **	48 (5)	52 (4)	55 (5)	61 (6)	54 (7)	34 (3)	37 (3)	39 (3)	44 (5)	39 (5)
Fat mass index, kg/m² ***	6.2 (1.5)	7.5 (1.3)	8.5 (1.4)	10.4 (2.1)	8.2 (2.2)	9.2 (1.3)	11.5 (1.0)	13.5 (1.0)	17.1 (2.5)	12.8 (3.3)
Lean mass index, kg/m² $	17.4 (0.9)	19.1 (0.4)	20.3 (0.4)	22.5 (1.5)	19.8 (2.1)	14.7 (0.6)	16.0 (0.3)	17.0 (0.3)	19.0 (1.3)	16.7 (1.8)

Conventions and exclusions as per Table 1. * Groupings of lean mass index are sex-specific quartiles of the baseline distribution.

Webtable 3: Correlation of markers of body composition in those aged 35-74 at recruitment and eligible for the prospective analyses

	WHR	BMI	FMI	LMI	Residual FMI	Residual LMI
BMI	0.45	Men				
	0.25	Women				
FMI	0.64	0.94	Men			
	0.29	>0.99	Women			
LMI	0.19	0.93	0.76	Men		
	0.18	0.99	0.98	Women		
Residual FMI	0.76	0.36	0.65	0.00	Men	
	0.51	0.15	0.22	0.00	Women	
Residual LMI	-0.45	0.33	0.00	0.65	-0.76	Men
	-0.46	0.08	0.00	0.22	-0.98	Women

WHR=waist-hip ratio, BMI=body mass index, FMI=fat mass index, LMI=lean mass index. Values below the diagonal are the Pearson correlation coefficients separately for men (top) and women (bottom).

Webtable 4: Predicted body composition and cause-specific vascular-metabolic mortality at ages 40-74 years - sensitivity analyses

	Men					Women				
		Death RR (95% CI) per 1kg/m² higher baseline level					Death RR (95% CI) per 1kg/m² higher baseline level			
Cause of death	No. of deaths	FMI	Residual FMI	LMI	Residual LMI	No. of deaths	FMI	Residual FMI	LMI	Residual LMI
**(a) After inclusion of participants with undiagnosed diabetes (no previously-diagnosed diabetes at recruitment, but glycosylated haemoglobin at least 6.5%)**										
Vascular										
IHD	425	1.11 (1.05, 1.16)	1.13 (1.05, 1.22)	1.12 (1.06, 1.18)	1.02 (0.94, 1.10)	344	1.09 (1.05, 1.13)	1.61 (1.34, 1.94)	1.13 (1.06, 1.21)	0.61 (0.44, 0.85)
Stroke	112	1.20 (1.09, 1.32)	1.49 (1.26, 1.76)	1.09 (0.98, 1.21)	0.78 (0.66, 0.92)	190	1.06 (1.01, 1.11)	1.45 (1.13, 1.87)	1.06 (0.97, 1.16)	0.58 (0.36, 0.93)
Other vascular	130	1.07 (0.98, 1.16)	1.25 (1.08, 1.46)	1.01 (0.92, 1.10)	0.80 (0.68, 0.93)	224	1.09 (1.05, 1.14)	1.12 (0.89, 1.41)	1.18 (1.09, 1.27)	1.02 (0.67, 1.55)
Subtotal: Any vascular	667	1.11 (1.07, 1.16)	1.21 (1.13, 1.28)	1.09 (1.05, 1.14)	0.93 (0.87, 0.99)	758	1.09 (1.06, 1.11)	1.42 (1.25, 1.61)	1.13 (1.08, 1.18)	0.69 (0.55, 0.87)
Metabolic										
Renal/Acute diabetic crisis	194	1.16 (1.08, 1.25)	1.19 (1.07, 1.34)	1.11 (1.03, 1.20)	0.97 (0.86, 1.08)	248	1.18 (1.13, 1.24)	1.17 (0.94, 1.46)	1.33 (1.22, 1.44)	1.55 (1.05, 2.30)
Hepatobiliary	313	1.09 (1.04, 1.16)	1.15 (1.05, 1.26)	1.04 (0.98, 1.10)	0.96 (0.87, 1.06)	250	1.15 (1.10, 1.21)	2.07 (1.65, 2.58)	1.26 (1.16, 1.36)	0.37 (0.25, 0.55)
Subtotal: Any metabolic	507	1.12 (1.07, 1.17)	1.17 (1.09, 1.25)	1.07 (1.02, 1.12)	0.96 (0.89, 1.04)	498	1.17 (1.13, 1.21)	1.60 (1.37, 1.87)	1.29 (1.22, 1.37)	0.72 (0.54, 0.94)
All vascular-metabolic	1174	1.12 (1.08, 1.15)	1.19 (1.13, 1.25)	1.08 (1.05, 1.11)	0.95 (0.90, 0.99)	1256	1.12 (1.10, 1.14)	1.50 (1.36, 1.65)	1.19 (1.15, 1.23)	0.70 (0.59, 0.83)
**(b) After restricting to never smokers only**										
Vascular										
IHD	76	1.04 (0.92, 1.17)	1.16 (0.97, 1.40)	1.03 (0.91, 1.16)	1.01 (0.85, 1.21)	204	1.09 (1.04, 1.14)	1.40 (1.10, 1.79)	1.13 (1.04, 1.23)	0.71 (0.46, 1.10)
Stroke	16	1.18 (0.93, 1.50)	1.22 (0.67, 2.24)	1.16 (0.89, 1.50)	0.76 (0.47, 1.22)	124	1.12 (1.06, 1.19)	1.59 (1.16, 2.17)	1.17 (1.05, 1.31)	0.69 (0.39, 1.23)
Other vascular	23	1.19 (0.97, 1.45)	1.87 (1.27, 2.76)	1.07 (0.87, 1.31)	0.57 (0.38, 0.86)	135	1.10 (1.04, 1.16)	1.47 (1.07, 2.00)	1.18 (1.07, 1.30)	0.84 (0.48, 1.46)
Subtotal: Any vascular	115	1.11 (1.01, 1.22)	1.35 (1.16, 1.58)	1.05 (0.96, 1.16)	0.87 (0.75, 1.01)	463	1.10 (1.07, 1.14)	1.45 (1.23, 1.70)	1.16 (1.10, 1.22)	0.76 (0.56, 1.02)
Metabolic										
Renal/Acute diabetic crisis	27	1.23 (1.03, 1.48)	1.16 (0.88, 1.52)	1.19 (0.98, 1.44)	1.16 (0.87, 1.55)	144	1.20 (1.13, 1.27)	0.96 (0.71, 1.30)	1.35 (1.22, 1.50)	2.68 (1.57, 4.58)
Hepatobiliary	55	1.18 (1.04, 1.35)	1.11 (0.89, 1.39)	1.16 (1.01, 1.34)	1.05 (0.85, 1.31)	151	1.17 (1.11, 1.24)	1.68 (1.25, 2.25)	1.31 (1.19, 1.45)	0.63 (0.37, 1.06)
Subtotal: Any metabolic	82	1.21 (1.08, 1.35)	1.13 (0.95, 1.34)	1.17 (1.05, 1.32)	1.09 (0.92, 1.30)	295	1.18 (1.14, 1.23)	1.29 (1.05, 1.58)	1.33 (1.24, 1.43)	1.24 (0.86, 1.80)
All vascular-metabolic	197	1.15 (1.07, 1.24)	1.25 (1.12, 1.41)	1.10 (1.03, 1.19)	0.96 (0.86, 1.07)	758	1.13 (1.11, 1.16)	1.39 (1.22, 1.57)	1.22 (1.17, 1.28)	0.92 (0.73, 1.16)

Conventions, main exclusions and analyses as per Figure 2; but analyses are for cause-specific mortality endpoints (WebTable 1), and further include or exclude participants as described. Results displayed in the table are the average mortality RR (95% CI) per 1kg/m² higher baseline level.

Webtable 5: Comparison of the 'informativeness' of different body composition indices (measured only once) for cause-specific mortality rates at ages 40-74 years

	Relative informativeness* for									
	IHD mortality		Stroke mortality		Any vascular mortality		Any metabolic mortality		Vascular-metabolic mortality	
**Body composition index**	**Men**	**Women**	**Men**	**Women**	**Men**	**Women**	**Men**	**Women**	**Men**	**Women**
Body Mass Index	100%	100%	100%	100%	100%	100%	100%	100%	100%	100%
Waist-hip Ratio	66%	62%	86%	265%	103%	78%	254%	26%	148%	45%
Fat Mass Index	78%	121%	179%	133%	101%	118%	211%	104%	137%	110%
Lean Mass Index	78%	74%	28%	41%	54%	78%	47%	83%	51%	81%

*Informativeness of the given index (as indicated by the age-stratified and confounder-adjusted χ^2 statistic relating it to cause-specific mortality), as a percentage of the informativeness of the body mass index. Estimates of the informativeness of each body composition index are adjusted for age, district of residence, educational level, smoking status, alcohol intake and leisure-time physical activity.
